# Observing and modeling long-term persistence of *P. noctiluca* in coupled complementary marine systems (Southern Tyrrhenian Sea and Messina Strait)

**DOI:** 10.1038/s41598-022-18832-2

**Published:** 2022-09-01

**Authors:** A. Bergamasco, A. Cucco, L. Guglielmo, R. Minutoli, G. Quattrocchi, R. Guglielmo, F. Palumbo, M. Pansera, G. Zagami, M. Vodopivec, A. Malej, A.Granata

**Affiliations:** 1 Institute of Marine Sciences, National Research Council (CNR-ISMAR), Section of Venice, Castello 2737/F, 30122 Venice, Italy; 2 Institute for the Study of Anthropic Impact and Sustainability in the Marine Environment, National Research Council (CNR-IAS), Section of Oristano, Località Sa Mardini - TorreGrande, 09170 Oristano, Italy; 3 Integrative Marine Ecology Department, Stazione Zoologica Anton Dohrn, Villa Comunale, 80121 Naples, Italy; 4 Department of Chemical, Biological, Pharmaceutical and Environmental Sciences, University of Messina, Viale F. Stagno d’Alcontres, 98166 Messina, Italy; 5 Department of Research Infrastructures for Marine Biological Resources, Marine Organism Taxonomy Core Facility, Ischia Marine Centre, Stazione Zoologica Anton Dohrn, Punta San Pietro, 80077 Ischia, Naples, Italy; 6 Administrative Secretary of the Marine Biotechnology Department, Stazione Zoologica Anton Dohrn, Villa Comunale, 80121 Naples, Italy; 7 RIMAR Department, Environmental Monitoring and Analysis, Stazione Zoologica Anton Dohrn, Villa Comunale, 80121 Naples, Italy; 8 National Institute of Biology, Marine Biology Station Piran, Fornace 43, 6330 Piran, Slovenia

**Keywords:** Ecology, Ecosystem ecology, Marine biology

## Abstract

In the Mediterranean Sea, the Strait of Messina (MS) is a very peculiar area, connecting highly different regions and representing a privileged observatory for an early comprehension and assessment of ecosystems shifts. It is hypothesized that the outbreaks observed near the coast of many sites in the Mediterranean Sea may be the result of transport of permanent populations of *P. noctiluca* in pelagic waters to the coast, caused by specific hydrodynamic conditions. By both visual observations and numerical experiments our objective is twofold: (A) to help clarify whether the basin of the Aeolian Islands Archipelago (AIA), in the Southern Tyrrhenian Sea (STS), may be the site from which large populations of *P. noctiluca* are transported to the MS, and (B) to evaluate whether the upwelling turbulent system of the MS can be an energetic opportunity for this species. It should offer a rich habitat without jeopardizing the overall survival of the population, that is subject to stranding due to strong currents. Although very different, the two involved ecosystems (AIA and MS ) are complementary for the success of *Pelagia noctiluca* life cycle. Outputs obtained by coupling the 3D hydrodynamic model (SHYFEM) with a Lagrangian particle tracking model support the hypothesis of a connectivity between these two ecosystems, particularly in the first half of the year, indicating the coastal areas around the AIA as potential optimal source location for *Pelagia* larval stages. We support the very attractive hypothesis that two connected systems exist, the former one favours *Pelagia*'s reproduction and acts as a nursery and the latter favours its growth due to higher productivity. We speculate that the reproductive population of the AIA is not permanent, but is renewed every year by individuals who have fed and quickly grown in the MS and who are passively transported by downwelling along canyon "corridors".

## Introduction

Gelatinous zooplankton are found throughout the world's oceans, but their species composition and abundance vary at local rather than global scales. The biomass of gelatinous zooplankton (Cnidaria, Ctenophora, Thaliacea) is greatest in productive coastal regions and is mainly related to sea surface temperature, dissolved oxygen, distance from shore, and primary production^1^. However, the occurrence of jellyfish in a wide range of environmental conditions suggests that they can adapt to different ecological niches. Despite the lack of scientific consensus on global long-term jellyfish trends^2^, their multiple negative impacts on human enterprises and ecosystem services^3^ are of concern^4^. Pelagic stages of Scyphozoa (scyphomedusae), particularly *P. noctiluca*, have been most commonly associated with negative impacts on coastal tourism, fisheries and aquaculture in the Mediterranean Sea^5,6^.

Unlike most scyphozoans, which have a metagenic life cycle with perennial polyps that require available substrate for attachment, *Pelagia noctiluca* is holoplanktonic. It inhabits both coastal waters and open oceans and is widespread in warm and temperate regions of the Atlantic and Pacific oceans^7,8^.

The success of their population in the Mediterranean Sea is reflected in their widespread distribution and increasingly frequent reports of blooms (outbreaks) in some coastal areas^9^. Marambio et al.^10^ consider *P. noctiluca* as the most common scyphozoan species in the recent review of jellyfish blooms in the western and central Mediterranean basins. Outbreaks have also been reported in the eastern Mediterranean along the coasts of Syria^11^, the Aegean Sea^12^ and Tunisia^13^. In contrast to the 1970s, 1980s and early 2000s, when *Pelagia* blooms occurred in the northern Adriatic Sea^14^, outbreaks in the last decade have been restricted to the central and southern Adriatic^15^.

Currently, *Pelagia* blooms in the western and central Mediterranean appear to be more common than observed until 1990^12,16–22^. Climatic factors such as North Atlantic Oscillation^23–26^ and especially the influx of Atlantic Surface Water (AW) in the Mediterranean Sea^27^ influence the temporal fluctuations of *P. noctiluca* in this area.

The hypothesis is that nearshore blooms in the Mediterranean Sea could arise from permanent *P. noctiluca* populations drifting from offshore waters to the coast due to specific hydrodynamic conditions^28,29^. Suitable winds and currents could favour the accumulation of specimens and thus the development of blooms, especially in semi-enclosed areas such as bays and harbours^13,21^. Models have been used to infer connectivity between different Mediterranean Sea subregions and forecast *Pelagia* transport from the pelagic zone to the coasts with subsequent stranding^9,22,30,31^.

Population connectivity is central to their viability^32^. Ecological connectivity at the relevant time scale, conferring stability to the overall demographic system, may be crucial for short-lived pelagic species. Spatio-temporal dynamics and reconstruction of putative source-sink locations are more straightforward for meroplanktonic scyphomedusae when the source location, i.e., benthic polyp habitats, are known^33^. Otherwise, assumptions about preferred habitat characteristics^34^ and potential refugia must be made, especially for holoplanktonic species.

Submarine canyons, geomorphic features of continental margins, are seascapes that provide specific pelagic habitats. They are characterized by diverse and complex hydrodynamics and have important ecological roles^35^. Although still poorly studied, the inventory of canyons in the Mediterranean Sea has been compiled^36^. Recently, it has been suggested^37^ that submarine canyons may be critical refugia for the Mediterranean *P. noctiluca*. Field observations of blooms in the proximity of canyons along the Catalan coast^9,38^ support this hypothesis. "Slope index", the distance from the nearest marine canyon, was included as a predictor in habitat models^10^. Slope index was a significant predictor of potential *P. noctiluca* blooming areas for the northern Catalan coast, the Tunisian coast near Bizerte, the Ligurian Sea, the Strait of Sicily and the Southern Tyrrhenian Sea (STS) near the Aeolian Islands Archipelago (AIA)^10^.

This work focuses on the connectivity of populations in the STS (AIA) and the Strait of Messina (MS), combining data from field observations of *P. noctiluca* spatial distribution, stranding events, and numerical modelling. The modelling experiments were built on demographically relevant time scales consistent with previous life expectancy and reproduction findings. The average lifespan of holoplanktonic *P. noctiluca* was estimated to be nine months^39^. In the Mediterranean Sea, *P. noctiluca* spawns throughout the year^40^, with two seasonal peaks in spring and autumn^41^. The AIA and the MS provide complementary habitat qualities for *P. noctiluca*. The AIA has already been reported as a preferred area for the reproduction of *P. noctiluca* and formation of outbreaks^9^. The particular geomorphological and hydrological features of the MS have a remarkable influence on plankton production^42,43^ and create a specific habitat for *P. noctiluca* growth^44^.

This article aims to reveal the seasonal variability of *P. noctiluca* and its stranding events and unveil the connectivity between two western-central Mediterranean areas using high-resolution hydrodynamic modelling and Lagrangian particle tracking. We hypothesize that the STS and the MS act as a coupled system that enables the successful long-term maintenance of *P. noctiluca* populations. The latter is more productive, favours growth, and is the source of mature individuals who come back to the former that in turn provides better conditions for spawning and early development. Such a concept of interconnected systems may have broader applications also in other geographic areas.

## Material and methods

### Study sites

#### Aeolian Islands archipelago

**Figure 1:**
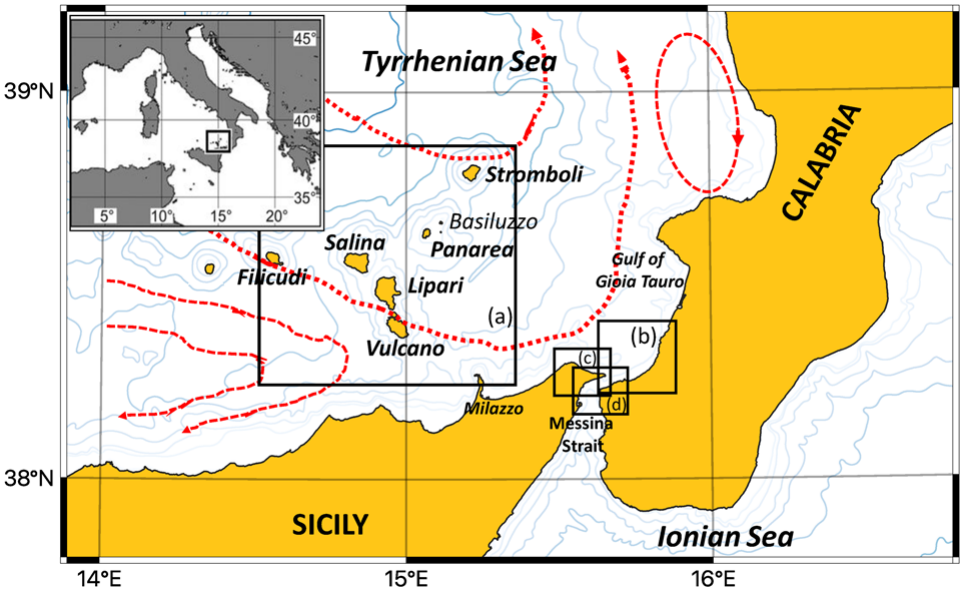
The eastern sector of the Southern Tyrrhenian Sea: boxes indicate the following sub-regions (**a**) Aeolian Island Archipelago; (**b**) Calabrian coast; (**c**) Sicilian coast; (**d**) Messina Strait. The main paths of surface circulation are depicted: the stream of AW flowing geostrophically and the anticyclonic recirculation cells. Map was generated with QGIS v. 3.22.6-Białowieża (https://qgis.org/it/site/) under GNU General Public License. Coordinate Reference System: EPSG:4326 (WGS84).

AIA is located in the SE Tyrrhenian Sea. The STS is characterized by a surface west–east flow of modified Atlantic Water (AW). AW enters the area near the northern Sicilian coast, forming a broad cyclonic eddy that carries water towards the northern part of the sub-basin and moves geostrophically along the Italian coast^45–48^. According to recent findings on the variability of the surface circulation of the STS^49^, the AW current, while moving cyclonically northward, forms broad meanders around several anticyclonic cells located along the Italian coast during winter and spring (Fig. 1). The first anticyclonic cell is off the Sicilian coast, the second in the Gulf of Gioia Tauro, and the others are roughly off Naples and Rome. On a more local scale, a cyclonic subcell^50,51^ drags the AW out of the broad main eddy to enter the AIA basin from the northwest and flow southeastward toward the coasts of Sicily and MS. The estimated mean flows may be of the order of 5–10 cm s^-1^. As the summer season approaches, the aforementioned anticyclonic cells of the coastal recirculation along the Italian peninsula appear to shift offshore, towards the west. The winter flow pattern of AW collapses due to the weakening of the cyclonic wind stress curl, which reverses in summer and becomes anticyclonic in the region^49^.

#### Strait of Messina

**Figure 2:**
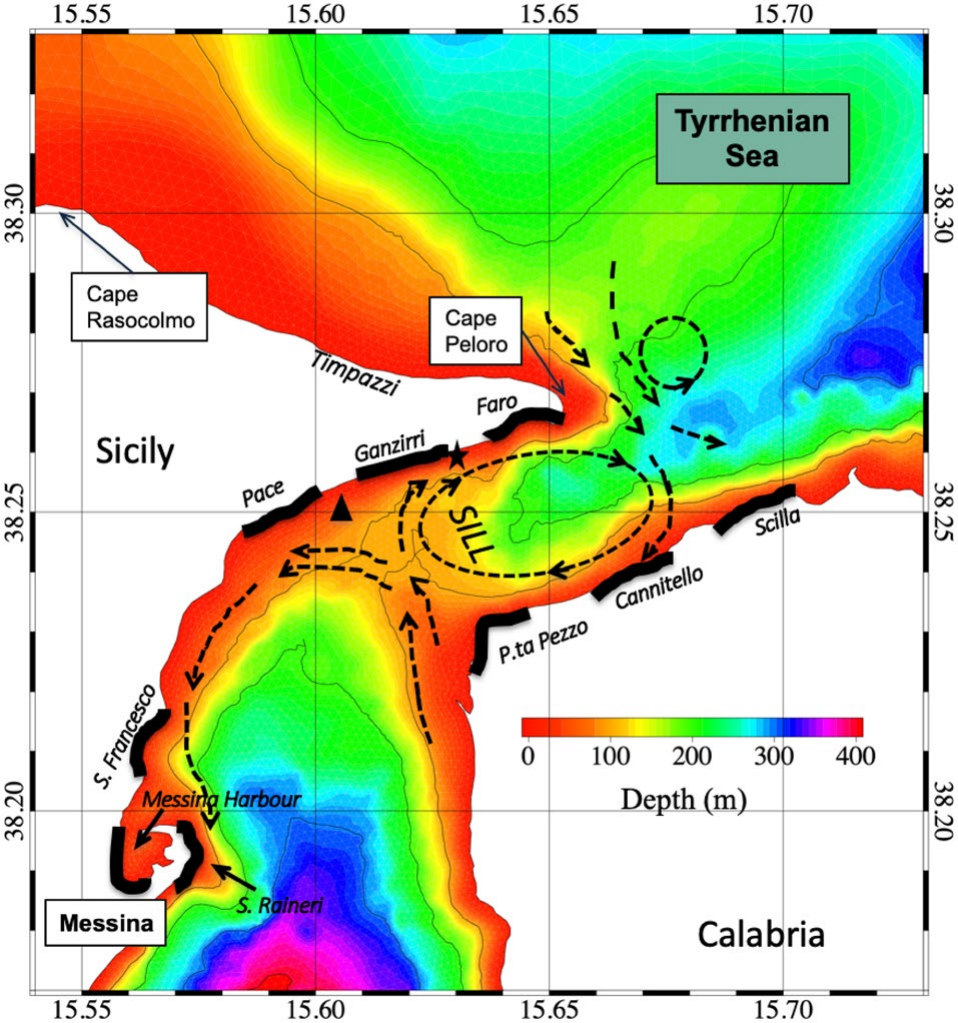
The Strait of Messina: the main morphological and bathymetrical features are indicated. The residual current pattern (redrawn from^52^) is superimposed (black arrows): (i) the advective eddy at the entrance of the Strait, in the lee side of Cape Peloro (ii) the anticyclonic recirculation pattern just north of the sill and (iii) the cyclonic residual circulation south of the sill can be recognized. The black star indicates the position of the ENERMAR-Kobold platform that provided water temperature near the seabed and current velocity data; the black triangle indicates the CTD station (T/S profiles). The 1-km-long stretches of coast monthly surveyed for jellyfish strandings are highlighted in black (six along the Sicilian coast; three along the Calabrian coast). Map was generated by SHYFEM v. 7.5 with the embedded visualization tool “shyplot” (https://github.com/SHYFEM-model/shyfem).

The MS separates Sicily from the Italian peninsula and connects the Ionian Sea in the south with the Tyrrhenian Sea in the north (Fig. 2). Detailed hydrographic information can be found in Vetrano et al.^48^ and Cucco et al.^52^ and references therein. From a hydrological point of view, the MS exhibits very relevant tidal currents driven by both barotropic and baroclinic processes, which depend on strong bathymetric forcings exerted mainly by the presence of a transverse sill (about 70 m deep) and a steep coastal morphology^53^. It has been reported that the maximum current intensity often exceeds 2 m s^−1^ in spring^54^. During the ebb phase ("scendente"), the Tyrrhenian surface water is directed into the MS and moves southward, while during the flood phase ("montante"), the currents are directed northward. The interaction with the topography and bathymetry of the channel leads to the formation of advective eddies and strong horizontal current shears, generally located in the lee of the prominent capes (Cape Peloro, Sicily and P.ta Pezzo, Calabria)^54^. The residual tidal flow (generally around 0.2–0.3 m s^−1^) shows the formation of an anticyclonic recirculation area just north of the sill with residual currents towards the Tyrrhenian Sea along the Sicilian coast (from Ganzirri to Cape Peloro) and towards the Ionian Sea along the Calabrian coast (Fig. 2).

The particular geomorphological characteristics of the MS lead to intense upwelling phenomena of Intermediate Ionian Waters in a specific area just south of the sill^55^. In contrast to other parts of the Mediterranean Sea, a stable thermocline can't develop in the MS, due to strong tidal currents and upwelling^56^. These conditions have a remarkable impact on the abundance and structure of planktonic communities^43,56–58^. Indeed, the total primary production rate has been estimated to range from 0.22 to 1.56 mg C m^−2^ h^−1^^42^. This stimulating conditions lead to great biological richness and biodiversity in this ecosystem, as evidenced by the phytoplankton, zooplankton and fish communities^43,57^.

### Habitat features in the Strait of Messina

To characterise the physical structure of the water column in this dynamic region, repeated vertical CTD casts were made down to 100 m depth (Fig. 2). Profiles were collected near the sill (38° 14′ 36ʺ N 15° 36′ E) every two weeks for 15 months from February 2003 to May 2004. A SeaBird 911 equipped with an altimeter was used. Overall twenty-nine available vertical profiles were analysed^59,60^.

In addition, automatic measurements from the Kobold ENERMAR platform anchored off the village of Ganzirri (38° 15′ 31ʺ N, 15° 37′ 40ʺ E) were used to characterize the water temperature at the seafloor (22 m depth) and the current regime near the Sicilian coast (Fig. 2). Water temperature measurements were collected during 2009–2010 (every 15 min from October 2009 to June 2010, every 1–6 h from June to October). Currents were measured every 15 min using a 3D ADCP (Nortek Aquadopp). In total, approximately 20,000 valid values of current intensities were acquired from October 2008 to May 2009^61^.

### Visual observations in coastal and open waters of the study areas

The *Pelagia* blooms, and spawning events in the Aeolian Archipelago have been documented and filmed over the past 18 years by diving expert Dario Lopes (pers. comm.). To observe the jellyfish larval stages (planulae and ephyrae) during blooms, samples were collected using a 2-L Niskin bottle, while juvenile and adult specimens were collected using a hand net. Collected individuals were observed and measured within 1 h using a stereomicroscope.

**Figure 3:**
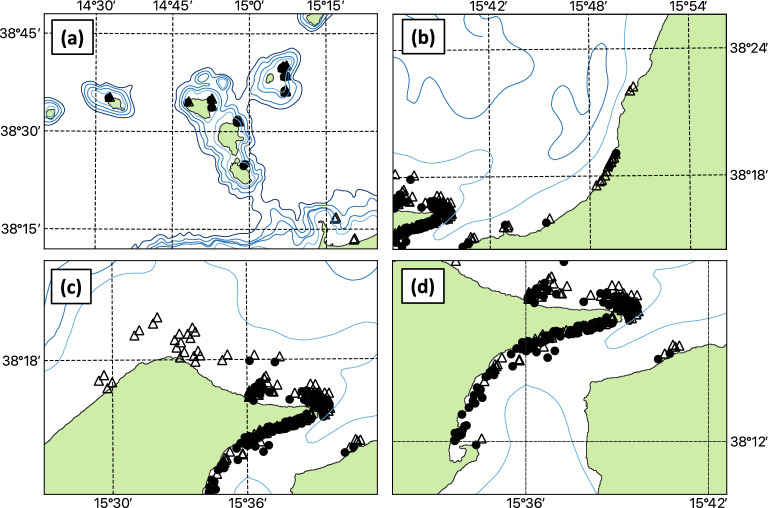
Visual observation effort in coastal and open waters sub-regions of the study area during March 2015–January 2016: (**a**) Aeolian Island Archipelago; (**b**) Calabrian coast; (**c**) Sicilian coast; (**d**) Messina Strait. Reported observations of *Pelagia noctiluca* in January–June (black dots) and July-December (empty triangles) are indicated. Maps were generated with QGIS v. 3.22.6-Białowieża (https://qgis.org/it/site/) under GNU General Public License. Coordinate Reference System: EPSG:4326 (WGS84).

Monitoring of jellyfish presence and abundance was carried out almost daily from the beginning of March 2015 to January 2016 on board the Tuccoli T25 Sports Fishing "Suerte", a 7.06 and 2.47 m long and wide boat used for swordfish fishing in the Strait of Messina and adjacent areas (Fig. 3). Each survey started from the Tyrrhenian Sicilian coast (38° 16.516ʹ N, 15° 35.998ʹ E) early in the morning (6 am) and ended around 7 pm on the same day. The repeated outward and return trips lasted 4–6 h each and included alternately the MS, the Tyrrhenian coastal stretches of Sicily (from Messina to Milazzo) and Calabria (from Scilla to Gioia Tauro), and the region around AIA on days with good weather conditions (smooth sea, with waves, generally 0.2 m, light or weak breeze). The boat travelled at an average speed of 2 knots, which allowed good observations of the sea surface down to a depth of 5 m. Observations were made from the deck of the boat at a height of about 2 m above the sea surface, using a spotlight after sunset. An observation area of about 40 square metres was marked out around the boat. Currents, wind and sea state were noted during the cruise.

A total of 181 surveys were conducted on different dates in the patrolled areas (Fig. 3). The 412 reported jellyfish occurrences were estimated by eye count and reported as number categories from 0 to 3: 0 no individuals observed, 1 for 1–10 individuals, 2 for 10–100 individuals and 3 for more than 100 individuals. Only in two cases were specific events with several hundred individuals were observed and marked as 'large aggregation'. These classes of abundance were considered sufficient to describe the distribution of *P. noctiluca* in the study area and are consistent with the earlier approach^62^.

To integrate available information within the Strait of Messina, data on the presence of jellyfish published by Rosa et al.^21^ and the Jellywatch CIESM monitoring from 2014 to 2015 (http://www.ciesm.org/marine/programs/jellywatch.htm) were included. Other validated information obtained through the Citizen Science program Meteomedusa, reports from well-known naturalists (youtube, video clips), beach guards, fishers or news from the local press (e.g. TempoStretto: info@tempostretto.it) were summarised.

### Strandings of Pelagia in the Strait of Messina

Stranding events of epi-, meso- and bathy-pelagic organisms along the Sicilian and Calabrian coasts of the MS are a very common phenomenon observed for a long time^63–66^. This allowed us to identify the oceanographic, meteorological and geomorphological causes and consequently to select the most suitable beaches for the collection of stranded *P. noctiluca*.

Specifically, a survey of *P. noctiluca* abundance was conducted along the coast of the MS from March 2015 to February 2016, based on two surveys per week at each site. The observations were carried out along the following selected beaches (from north to south, Fig. 2) (a) Sicilian coast: Faro, Ganzirri, Pace, S. Francesco, and S. Raineri (b) Calabrian coast: Scilla, Cannitello and P.ta Pezzo. The survey took place in the morning and afternoon and essentially involved a careful inspection for *Pelagia* occurrences along the shoreline. Information on wind and current directions and weather conditions were recorded on the sampling days. In addition, the presence of *Pelagia* was also recorded within Messina Harbour, previously identified as a preferred location for large accumulations of floating debris.

In particular, about two stranding berms could be distinguished, of which the one farthest from the shore was characterized by the presence of pumice of the AIA, on the surface of which are barnacles, tubular annelids, and exceptionally, in the tiny cavities, small lamellibranchs. *Pelagia* was more commonly found along a berm closer to the shoreline, characterized by the presence of various pelagic organisms, and formed mainly in calm or slightly rough seas. For these reasons, beach sections approximately 1-km-long and 1-m-wide were surveyed by walking in both directions along identified stranding paths when there was more than one.

To determine the size/weight distribution of the stranded population, we calculated the average weight of jellyfish following Rosa et al.^21^. For specimens that were too damaged or dehydrated (especially in summer), we assumed that the stranded population was the same size as a reference specimen collected in the sea near the shoreline.

### The numerical model

A high-resolution 3D hydrodynamic model (SHYFEM, https://github.com/SHYFEM-model/shyfem) was used to test some of our hypotheses about the transport and stranding of *P. noctiluca*. The model resolves the system of shallow water equations integrated over each layer in their formulations with water levels and transport^67^. It is based on the finite element method and has been successfully used in several applications and case studies at Mediterranean Sea^68–72^. The unstructured mesh resolves the model equations at a different spatial resolution, which is particularly useful when studying water circulation in both open ocean and coastal environments.

**Figure 4:**
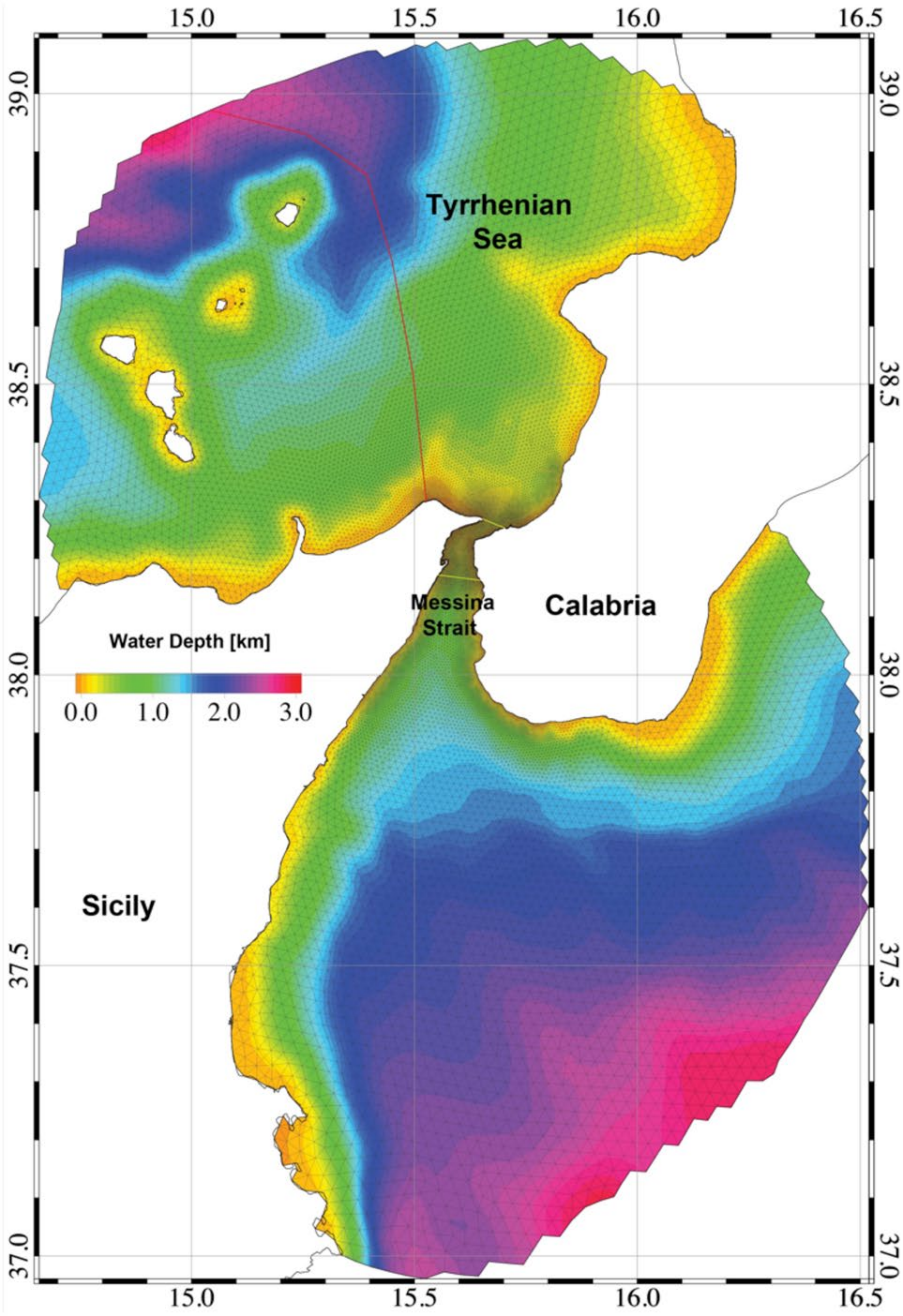
The model computational domain as represented by the finite element mesh with bathymetric details. Red line delimits the seeding area for Experiment 1, yellow lines enclose the MS area where numerical particles were seeded during Experiment 2. Map was generated by SHYFEM v. 7.5 with the embedded visualization tool ”shyplot” (https://github.com/SHYFEM-model/shyfem).

In particular, SHYFEM was used in a previous study^52^ to reproduce in detail the 3D water circulation caused by tides, heat and salt fluxes, and meteorological forcing in the MS, the STS, and the northern part of the Ionian Sea. In this study, the same model setup was used^52^, including the model mesh (Fig. 4) and boundary conditions, to reproduce the currents for the 2014–2015 period. This period was chosen since it includes most observations and measurements and is representative of recent years in terms of oceanographic and meteorological conditions^52^. The hourly model results (currents and winds) were used for the offline particle tracking simulations. The Particle Tracking Model (PTM) solves the advection and diffusion equations in a Lagrangian reference frame and allows the simulation of the path followed by active or passive numerical particles within the hydrodynamic model domain^73–76^.

The PTM uses the horizontal currents fields computed at a depth of 5 m to simulate the transport of the numerical particles and therefore neglects any vertical motion.

The contribution of wind to particle motion is very small and is assumed to be 4‰ of wind speed, as suggested by several modeling studies applying SHYFEM to reproduce the path followed by Lagrangian current- meters published in Western Mediterranean Sea^70,72,73^. In the Supplementary Note on model implementation, a detailed description of the model equation system and simulation setup is reported.

Three different modeling experiments were conducted:

*EXP1* The first experiment was focused on investigating the dispersion of the early life stages of jellyfish from the spawning regions around the AIA toward the MS. The approximation of a purely horizontal displacement is assumed to hold for *P. noctiluca* larval stages (mostly ephyrae) which preferentially remain in the surface layer^77^ and therefore their passive transport can be approximated as mainly driven by the surface currents. In this simulation, the coastal and shelf waters of the AIA and Northern Sicily in the STS (west of the red line in Fig. 4) were assumed as possible spawning areas and sources of numerical particle seeding. About 10,000 passive Lagrangian particles were homogeneously released in the selected area with semi-diurnal frequency and their transport by surface currents and winds was computed by PTM.

Our observations show that reproduction in Aeolian waters is most intense from mid-winter to late spring (February–June), with minor reproduction also occurring in late autumn^21,78^. Accordingly, numerical particle seeding in our model was performed from late January to early June. Hourly positions of each released particle were used in the analysis. Relative probability of entering the MS as well as travel time were calculated for each release position. Probability was calculated using the Normalized Source Index (NSI), a dimensionless index obtained by normalizing and averaging the ratio of the number of numeric particles that entered the MS to the total number of particles released for each element of the computational domain. The NSI describes the relative degree of connectivity between each element of the seeding regions with the MS.

*EXP2* The second simulation was conceived to evaluate the role of MS water circulation in promoting the retention and growth of *Pelagia* individuals. Horizontal passive transport of young/adult specimens by very strong barotropic currents dominates in the MS area so that their active vertical movements can be neglected to evaluate the trapping efficiency of the MS. Approximately 10,000 numerical particles were released uniformly with semi-diurnal frequency within the MS (between the yellow lines in Fig. 4). The trajectory of each particle was calculated and the relative abundance of particles within MS was estimated to determine the periods with the most favorable hydrodynamic conditions for trapping young *Pelagia* individuals within MS.

*EXP3* The third numerical experiment was designed to simulate stranding of *P. noctiluca* in MS during summer, when habitat conditions are most favorable for jellyfish growth and the highest number of strandings was observed. The approximation of a purely horizontal displacement is motivated by considering that landing of the jellyfish individuals is promoted and ruled by surface forcings only, such as the wind and surface currents. In this case, the seeding of the particles within MS (between the yellow lines in Fig. 4) was performed only during the summer months (June, July, and August), resulting in the release of approximately 8 million particles throughout the simulation. GIS processing was performed on modelled particle positions to count stranding events for each of the 5-km coastal sections (Fig. 15).

The numerical experiments were designed to investigate the possible path of early life stages of *P. noctiluca* specimens from their spawning sites to feeding grounds and to explore the fate of young individuals in the MS during specific periods of the year. The questions the chosen approach sought to answer were: "Where do they come from? When do they arrive at MS? How long does it take them to reach MS? How long do they stay?".

## Results

### Observational data and habitat features

#### Environmental data and habitat features in the Strait of Messina

**Figure 5:**
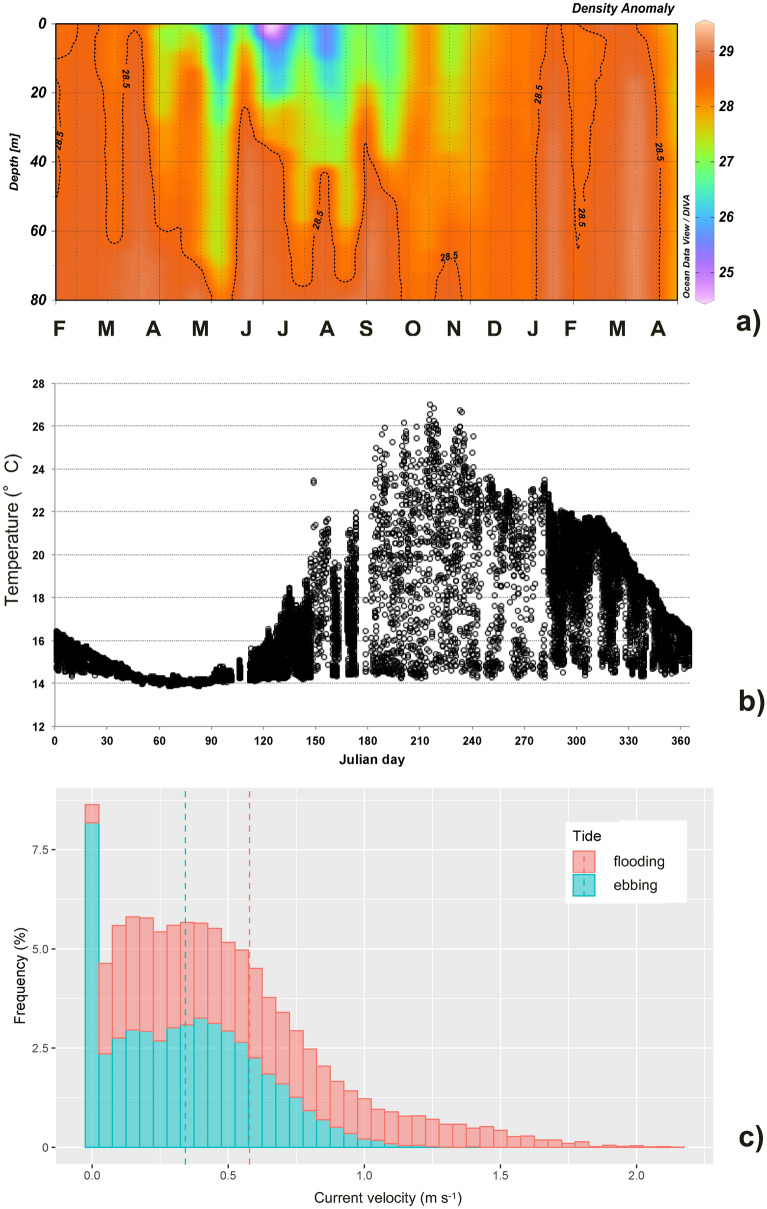
Habitat features relevant to *P. noctiluca* presence within the Messina Strait: (**a**) time series of bimonthly density anomaly profiles collected near the sill from February 2003 to April 2004: evidence of recurrent upwelling; (**b**) time series of water temperature at 20 m depth: optimal thermal range during the whole year; (**c**) frequency distribution of current intensity at 10 m depth near the Sicilian coast: active swimming very difficult and high risk of stranding.

Analysis of the CTD vertical data show strong upwelling of deeper Ionian water with T and S values within a narrow range (14.0 ± 1 °C and 38.5 ± 0.3, respectively), especially near the submarine sill. Taking the density anomaly of 28.5 σ_t_ as a upper limit of the deeper waters rising to the surface, Fig. 5a highlights their recurrent presence throughout the whole water column. This limit is confined below 40–50 m depth from early spring (March–April) to autumn (November) leading to the formation of a two-layer system, while during winter with the onset of thermal homogenization these dense waters invade the whole column. This phenomenon regularly draws nutrient-rich intermediate Ionian waters into the euphotic layer, benefiting the entire food chain.

A second important effect of this phenomenon can be observed by examining the dynamics of water temperature at a depth of 20 m (see Fig. 5b). In summer, strong temperature variations are observed due to the semi-diurnal tides that mix warmer Tyrrhenian surface water and colder upwelled Ionian water in the area. This contributes to the average temperature in the MS being lower than at the surface STS, where the temperature is often above 28 °C for several days in summer. On the other hand, the same phenomenon causes the water temperature in the Strait not to fall below 13.8–14 °C in winter. From a physiological point of view, the habitat in the MS is therefore in an optimal temperature range for *Pelagia*^79^, which is neither too hot in summer nor too cold in winter.

The third distinctive element of the habitat is the high current intensity which makes active swimming against current difficult for jellyfish. The currents dominate jellyfish transport which, given the turbulent regime, exposes jellyfish to significant stranding risks. The frequency distribution of the horizontal current intensity at 10 m depth is presented in Fig. 5c. It is interesting to note that less than 9% of time during slack the intensity is below 5 cm s^−1^, a threshold indicated by previous studies as the routine swimming speed for jellyfish^80^. Moreover, the intensity is on average about one order of magnitude above such value in both tidal phases (0.36 and 0.55 m s^−1^ respectively) and strongly asymmetric, being at least for 10% of time beyond 100 cm s^−1^.

#### Past documented *P. noctiluca* blooms and spawning events

Most of the documented and filmed intense blooms occurred between February and June. The first two documented blooms near Filicudi Island (see Fig. 1 for locations) are dated April 2008 and March 2009. One of the highest concentrations of *Pelagia* individuals was filmed near Vulcano Island in February 2010, while a large bloom was observed near Filicudi Island in May of the same year. In spring 2014, blooms were documented with videos and photos in both Salina (March) and Filicudi (March, May and June) (Lopez, pers. comm.). In particular, a dense bloom of *Pelagia* was documented in Filicudi on 2 June 2014, consisting of individuals in all developmental stages, from larvae to adults. The observed dense white cloud consisted of numerous planulae ranging in size from 0.34 to 0.36 mm. Among the larger specimens, adult jellyfish with a bell diameter of 70–80 mm were pink in colour (about 20%), while juvenile jellyfish (20–30 mm bell diameter) accounted for about 80%. Large aggregations of small (20–30 mm) and medium (50–60 mm) *Pelagia* specimens were observed in early July (3 and 16 July) near Basiluzzo Island (see Fig. 1 for location). In the same year, a massive occurrence of 2.5–3.5 mm ephyrae was noted during the winter months. Selected photos and videos of the reported mass events can be found in Supplementary Materials (V1).

These data confirm the assumption that AIA can be considered a natural ecosystem suitable for *P. noctiluca* reproduction from winter to late spring.

#### *P. noctiluca* occurrence and abundance patterns during 2015–2016

**Figure 6:**
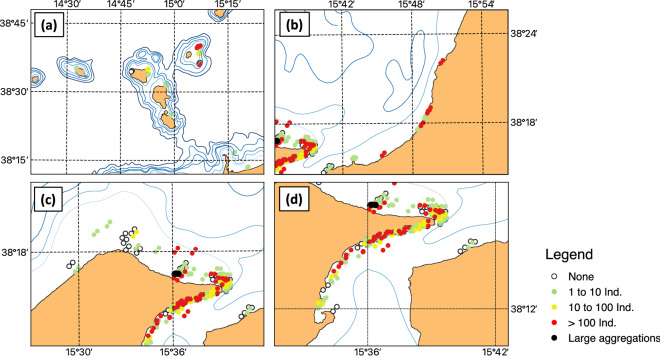
Presence and abundance patterns of *Pelagia noctiluca* specimens in the study area as observed during the 181 surveys of the fishing boat “Suerte” in the period March 2015–January 2016. Abundance classes are in agreement with^62^. Maps were generated with QGIS v. 3.22.6-Białowieża (https://qgis.org/it/site/) under GNU General Public License. Coordinate Reference System: EPSG:4326 (WGS84).

The boat surveys conducted in this study yielded nearly 2500 h of observations over a total distance of about 5000 nautical miles in the coastal and open waters of the region (Fig. 6). Direct observations confirmed the widespread occurrence of *P. noctiluca* at AIA and in the MS. Overall, *Pelagia* specimens were detected in about 80% of the surveys, and their abundance was high in almost 20% of the cases (> 100 ind., see red dots in Fig. 6). The occurrence was equally frequent in the MS and along the Tyrrhenian coast of Sicily (40–45% of reports each), while it was only occasional along the Calabrian coast. *P. noctiluca* were generally more abundant in the late morning (median 11:00 am) and rarely just before sunset, especially in summer.

**Figure 7:**
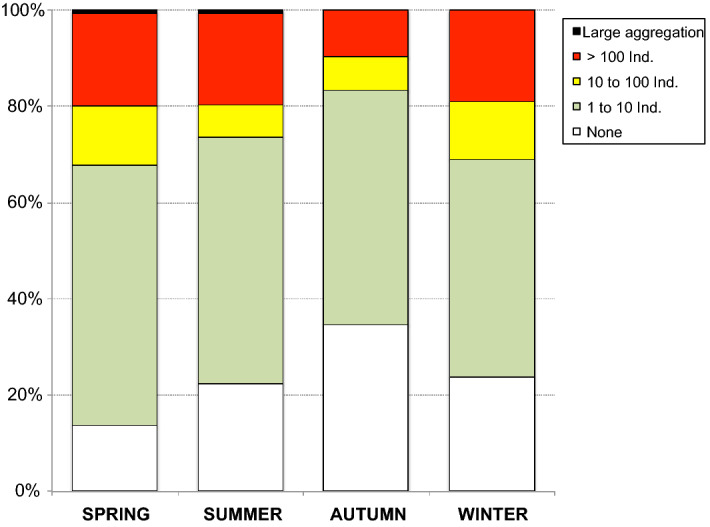
Visual observations of *Pelagia noctiluca* presence in the study area (March 2015–January 2016): Seasonal distribution of relative percentages of events in the five classes of abundance.

Higher densities of *P. noctiluca* were observed mainly in spring and summer (Fig. 7). Two events of large *Pelagia* aggregation were recorded in front of the Tyrrhenian beaches of Eastern Sicily in May and June, respectively (Figs. 6, 7). More specifically, the two blooms were observed on May 6th and June 21st 2015 in the afternoon (5 pm) (lat 38° 16′ 41″ N, lon 15° 36′ 10″ E) both in calm seas and without wind. In May the extremely high abundance of *P. noctiluca* was associated with huge numbers of the colonial hydrozoan *Velella velella*.

The highest frequency of *Pelagia* observed in the Cape Peloro area (see Fig. 2 for location) and in the surrounding areas of the STS occurred during prevailing NW winds (about 50% in the year), the relative frequency of which was highest in May and June (95% and 80%, respectively). In contrast, variable winds from NE and south prevailed from July onwards, with the relative frequency of NW winds being lowest in October (8%). According to the repeated observations, the large quantities of *Pelagia* observed along the Tyrrhenian coast from Cape Rasocolmo (see Fig. 2 for location) to Cape Peloro are often carried at the Timpazzi site, about 15 km west of Cape Peloro, by the ebbing ("scendente" N-S) current into the Strait of Messina, where they are driven back to the coast by the flooding ("montante" S–N) current. From 8 to 10 July large quantities of small and medium-sized *Pelagia* specimens were observed between Pace and Ganzirri (see Fig. 2 for locations) in the ebbing swirls, which disappeared almost entirely with the onset of the tide. Large quantities of *Pelagia* were also observed on April 7th at the entrance of Messina Harbour during the high tide phase, when the wind was blowing from the NW.

#### Beach strandings

**Figure 8:**
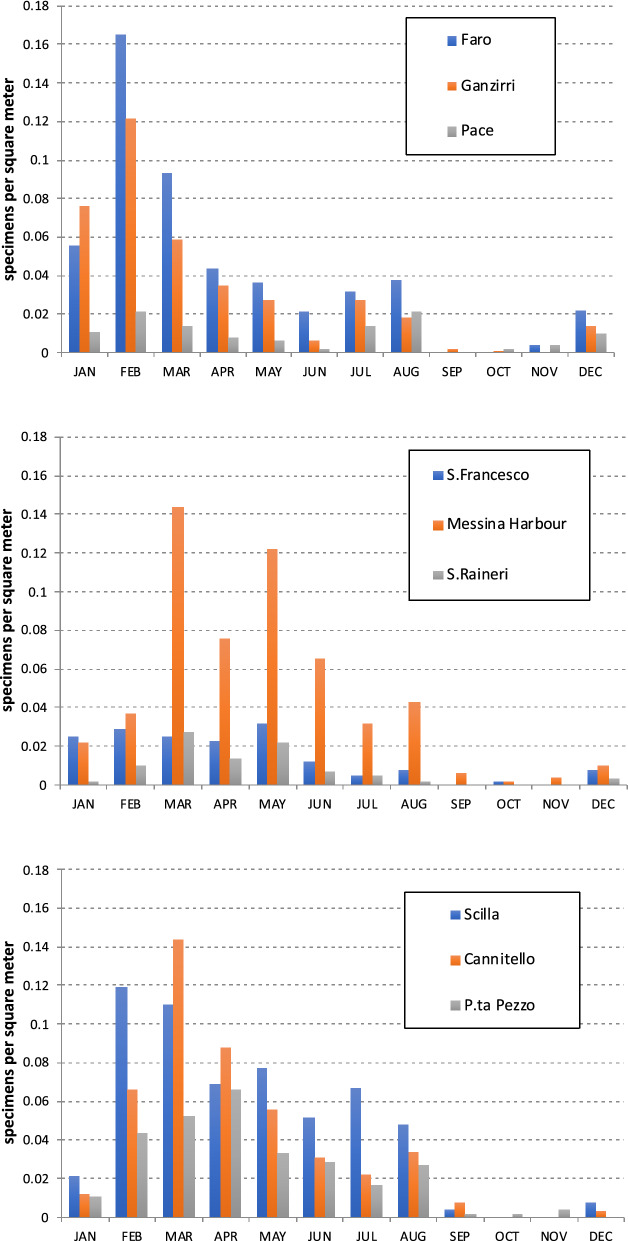
Jellyfish beach strandings: Upper and central panels: Sicilian coast (from N to S); Lower panel: Calabrian coast (from N to S).

Observations of stranded *Pelagia* were conducted from March 2015 to February 2016. A total of 3162 specimens were counted, of which 58% along the Sicilian coast (five beaches plus Messina Harbour, see Fig. 2) and 42% along the Calabrian coast (three beaches). On a yearly basis, Faro was the one with the highest number of stranded specimens (28%) among the Sicilian beaches, followed by Ganzirri (22%), S. Francesco (9%), Pace (6%) and S. Raineri (5%). Natural or artificial bays seemed to influence strandings: among the studied beaches, Faro and Ganzirri, where most of the strandings occurred, are the richest in such structures. The highest number of *Pelagia* specimens was observed along the docks of Messina Harbour (31%, Fig. 8). Among the Calabrian beaches, Scilla was the one with the highest number of stranded *Pelagia* (43%), followed by Cannitello (35%) and P.ta Pezzo (22%).

The relative frequency of stranded *Pelagia* on the Sicilian coast was high on days with easterly and southeasterly winds. At Faro and Ganzirri beaches, the highest number of stranded individuals was observed during southeasterly winds. Strandings at the southernmost beaches (e.g. S. Francesco) and along the Calabrian coast were mainly favoured by N/NE/NW winds. Strandings were highest at most sites during the winter and spring months (Fig. 8). They decreased in summer (July and August) and occurred in calm seas and light winds with variable directions. The stranded individuals collected were mostly damaged, colourless and usually without oral arms. The average percentage of bell diameter size classes recorded on the Sicilian and Calabrian beaches in the different seasons is presented in Table 1. The two largest stranded specimens with bell diameters of 102 mm and 124 mm were collected in January and February.

**Table 1:** Bell diameter of the stranded specimens of *Pelagia noctiluca* collected along the Sicilian and Calabrian coasts: seasonal trend of the relative incidence (in %) of each size class.

	Sicilian beaches				Calabrian beaches			
Bell diameter (mm)	20–30	30–50	50–70	70–90	20–30	30–50	50–70	70–90
Winter	5.9	42.9	48.8	2.4	9.7	50.2	39.4	0.7
Spring	0	7.8	68.8	23.4	0.4	17.2	64.7	17.7
Summer	0	3.4	73	23.6	0	3	77.3	19.7
Autumn	0	1.4	77	21.6	0	0	88.2	11.8

**Figure 9:**
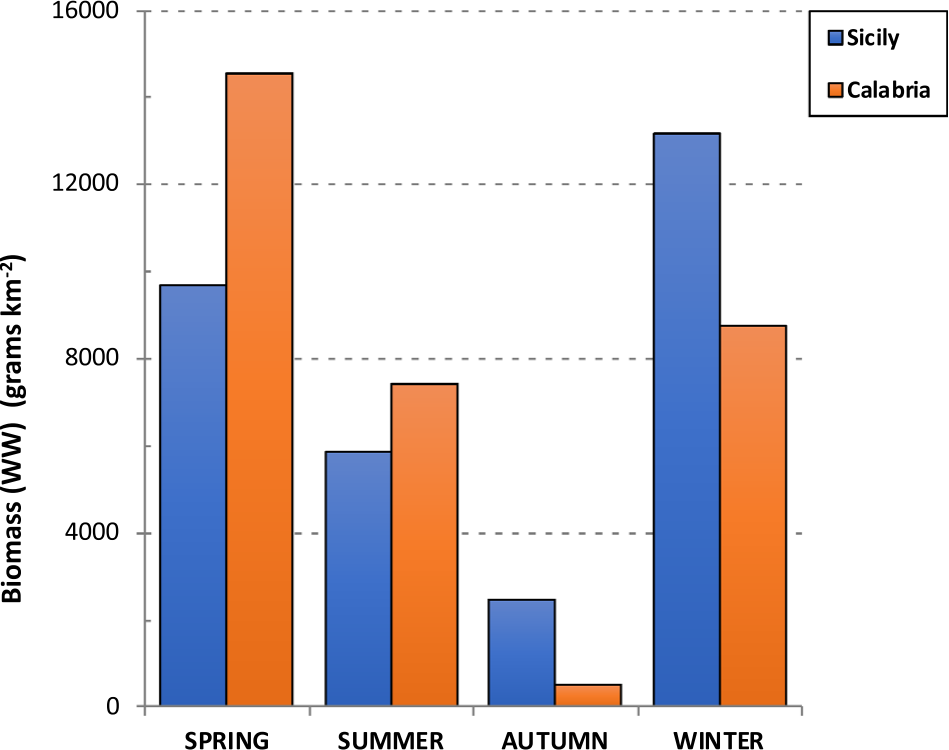
Seasonal trend of cumulated biomass of *P. noctiluca* stranded specimens along the coasts of Sicily (4 sites) and Calabria (3 sites) from March 2015 to February 2016.

The biomass of stranded *P. noctiluca* was estimated using the equation W = 0.0002 D^2.8786^ developed for the study area^21^. W is the total wet weight in grammes, and D is the bell diameter in mm. As shown in Fig. 9, the highest stranded *Pelagia* biomass was recorded on the Sicilian coast during the winter months (WW = 13,000 g, 42% of the total stranded biomass), with a decreasing seasonal trend until autumn, when the lowest value was recorded (WW = 2500 g, 8%). On the Calabrian coast, the highest biomass was obtained in spring (WW = 15,000 g, 47%), followed by similar values in winter and summer, to reach the lowest value in autumn (WW = 500 g, 2%). Although fewer beaches were considered on the Calabrian coast than on the Sicilian coast (3 versus 5 beaches), the cumulative biomass values were the same: 31,000 g WW (50%).

### The numerical experiments

#### From spawning to nursery (EXP1)

**Figure 10:**
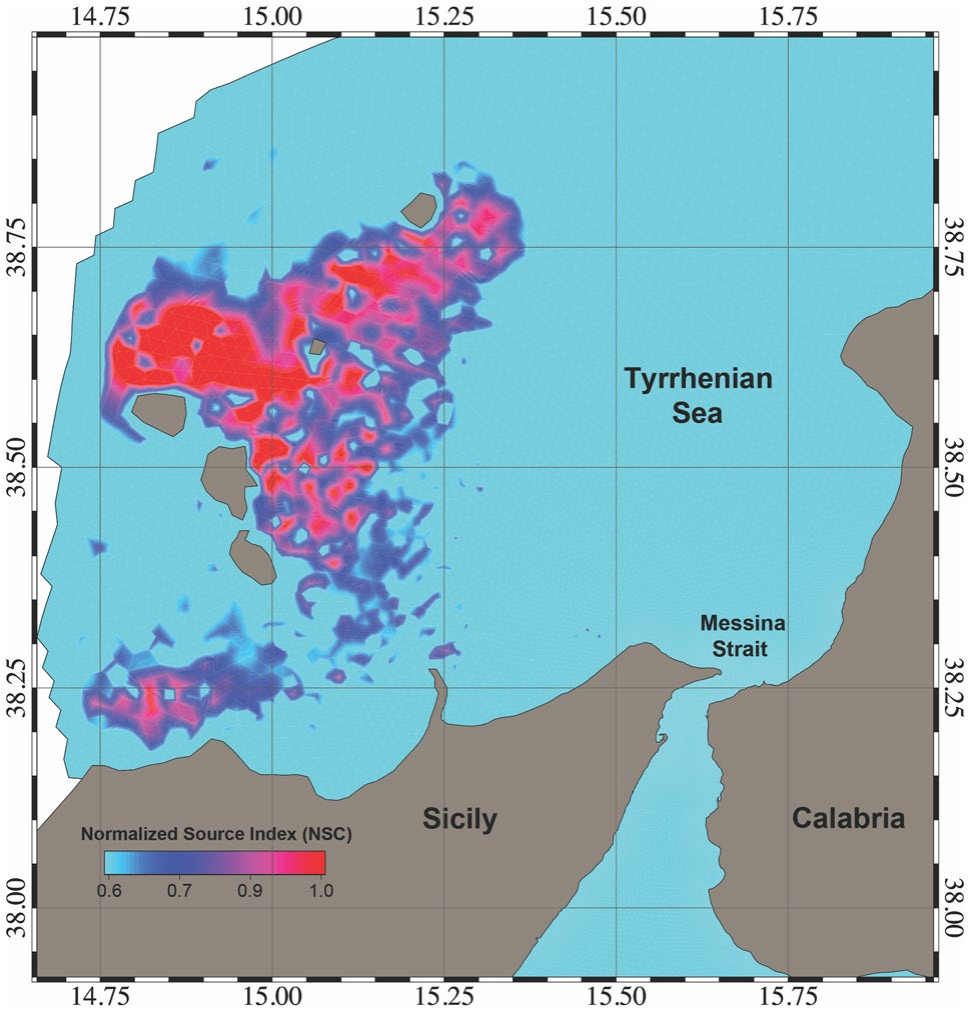
Distribution of the relative probability of arriving in the Strait starting from the selected spawning areas. The probability has been computed through the Normalized Source Index (NSI, hereafter), a non-dimensional index obtained by normalizing and averaging the ratio between the number of numerical particles entering in the Strait of Messina and the total number of particles released for each element of the computational domain. Map was generated by SHYFEM v. 7.5 with the embedded visualization tool ”shyplot” (https://github.com/SHYFEM-model/shyfem).

**Figure 11:**
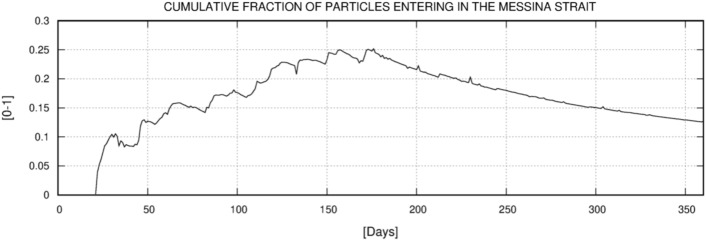
The cumulative ratio between the total amount of particles entering in the Strait and the amount of particles released within the spawning areas and still within the model domain. The results were post-processed and averaged on a daily basis in order to obtain a whole year dataset from the two-year simulation runs results. The time series of the ratio is reported as a function of the time.

**Figure 12:**
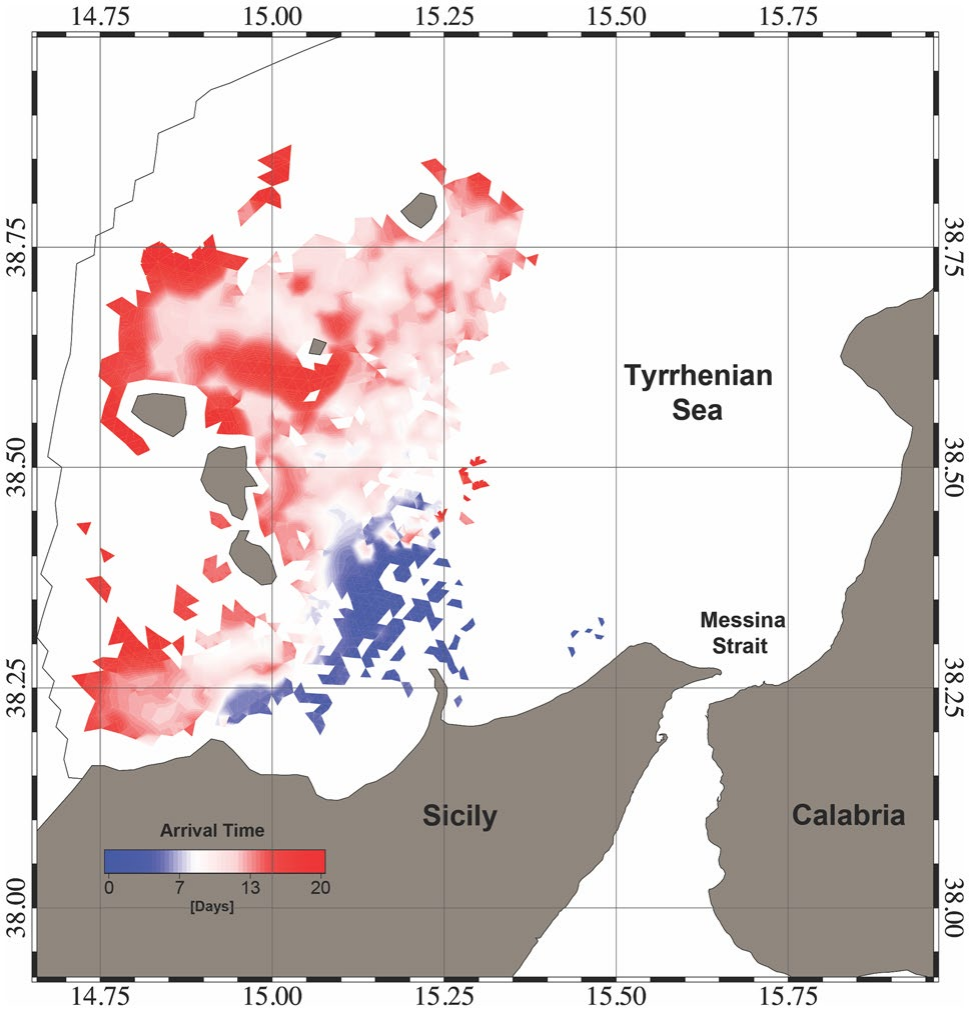
Distribution of average travel times to the Strait of the particles starting from the spawning areas with NSI higher than 0.6. These times correspond to the ages of the particles at the entering into the Strait of Messina. Map was generated by SHYFEM v. 7.5 with the embedded visualization tool ”shyplot” (https://github.com/SHYFEM-model/shyfem).

The first experiment results showed that two separate spawning areas contribute a relatively high number of particles to MS. These are clearly distinguishable in Fig. 10. The smaller area, located between the northern Sicilian coast and south of AIA, has a lower NSI with values between 0.7 and 0.8, while the larger area is located between the Aeolian islands of Salina, Stromboli and Vulcano and has an NSI above 0.9. The coastal waters around Lipari and Panarea have NSI values close to 1 and are therefore the most likely source of *P. noctiluca* in MS. The particle concentration timeline for MS is shown in Fig. 11. The first particles enter the MS about 25 days after the first release (January), and the maximum number is reached in June (day 170–175). After reaching the maximum concentration, the fraction of particles decreases until the end of the year with an almost constant rate and minimal disturbances. These results show the maximum influx of *P. noctiluca* in late spring and early summer and agree well with field observations. Transport time from spawning areas to MS was estimated based on particle age and is shown in Fig. 12. Particles from nearby areas reach MS within a few days, while others take up to 30 days. Particles from the areas with the highest NSI (Lipari and Panarea) reach MS in about 20 days.

#### Living in the Strait (EXP2)

**Figure 13:**
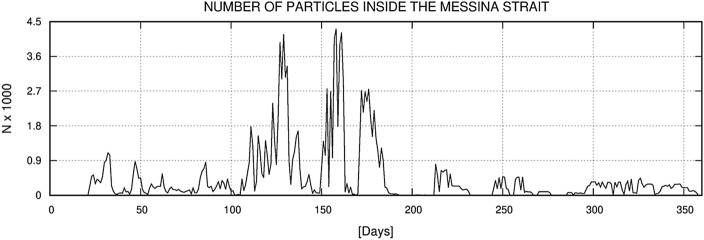
Daily amount of numerical particles inside the Strait throughout the year. A total of 20,000 particles were released every 24 h in the selected spawning areas around the Aeolian Island Archipelago.

**Figure 14:**
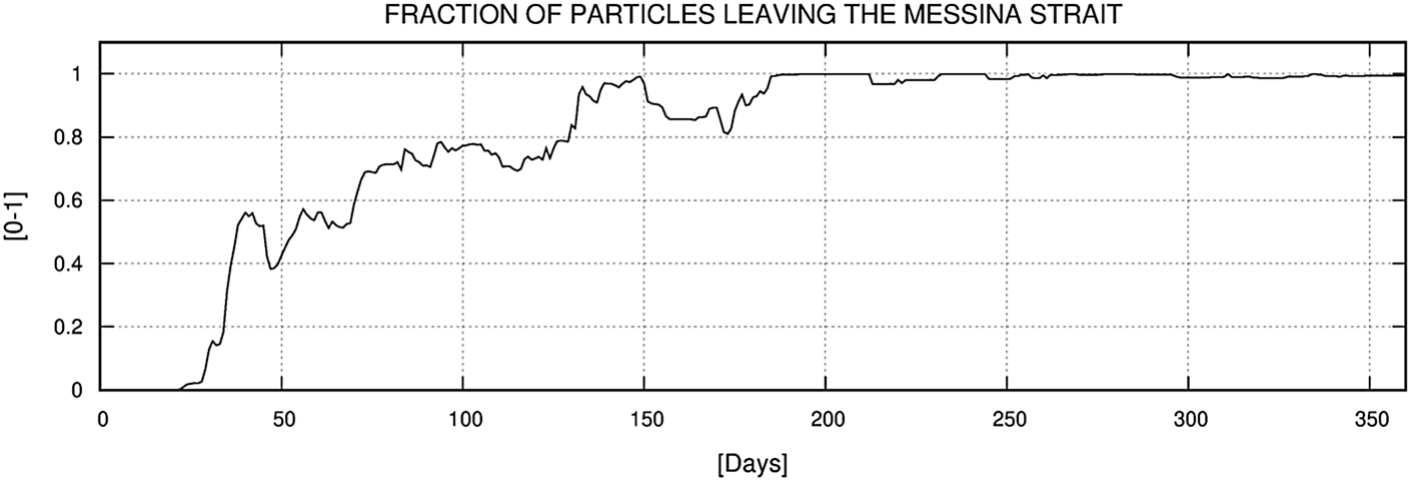
Evaluation of the retention efficiency of the Strait: ratio between the amount of numerical particles leaving the Strait to the Tyrrhenian Sea and the amount of numerical particles entering in the Strait.

In the second experiment, we investigated the suitability of hydrographic conditions for the retention of *P. noctiluca* in the MS. Figure 13 shows the number of particles in the MS for each day of the year. There are three distinct peaks in April, June and July. These are the periods that should provide the most favourable environment for *P. noctiluca* growth. Late spring and early summer also coincide with the time when the jellyfishes from AIA should arrive. We also evaluated the fraction of particles that end up in the STS (Fig. 14). The results suggest that particles are retained in the MS during the first part of the year, while the escape of particles into the STS is favoured thereafter.

#### Stranding (EXP3)

**Figure 15:**
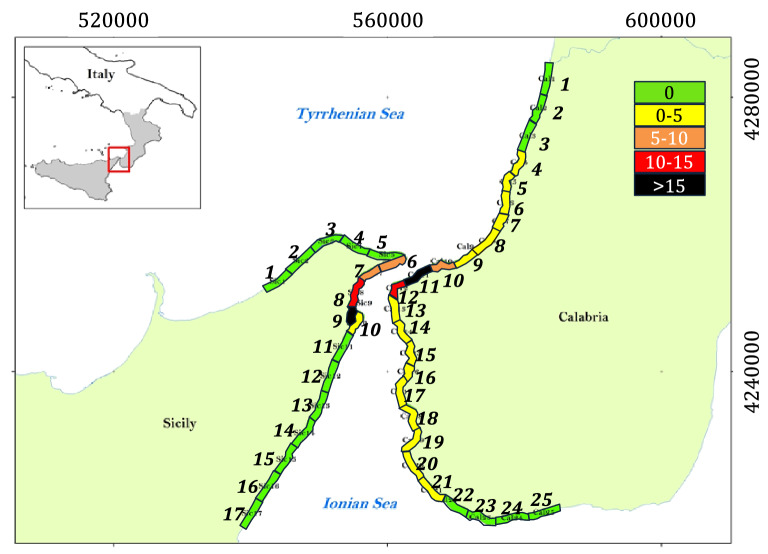
Modeled stranding shares (%) per each coastal element along the coasts of the Strait of Messina. Sicilian and Calabrian coasts were subdivided into 5 km-long stretches (clockwise from Sic1 to Sic17 for Sicily and counter clockwise from Cal1 to Cal25 for Calabria respectively) to count the stranding events. Along the Sicilian coast: Faro and Ganzirri beaches lay in Sic6; Pace beach lays in Sic7; S. Francesco beach lays in Sic8; Messina Harbour lays in Sic9; S. Raineri lays in Sic10. Along the Calabrian coast: Scilla beach lays in Cal10; Cannitello beach lays in Cal11, P.ta Pezzo beach lays in Cal12. Map was generated with QGis v. 3.22.6-Białowieża (https://qgis.org/it/site/) under GNU General Public License. Projected Coordinate System: EPSG:32,633 (WGS 84/UTM zone 33 N).

In Fig. 15, we plot the percentage of particles that are stranded at different parts of the coast. The stranding probability is only slightly higher on the Calabrian coast (53%) than on Sicilian Coast (47%). In Sicily, strandings are concentrated in the section between Cape Peloro (north) and Messina Harbour (south; see Fig. S1). Here, the shape of the coastline favours the trapping of numerical particles. In Calabria, most strandings occur between Scilla and P.ta Pezzo (about 40%). The remaining strandings extend over a much longer part of the coast than in Sicily.

#### Strandings comparison with in situ observations (EXP3)

We compared the results of the EXP3 experiment with observed *P. noctiluca* strandings. Considering the fragility of stranded individuals, they should not last much longer than one day, and therefore the number of strandings should be approximately equal to the daily stranding rate. We compared observed strandings for the same three months used in the simulation (June, July, and August = 92 days). According to our observational estimate, there were about 440,000 stranded individuals on the Calabrian beaches and about 280,000 stranded individuals on the Sicilian beaches during this period corresponding to a ratio between the individuals stranded along the two coastlines of about 1.5. Similar behavior was observed from the model simulations results: the ratio between the total particles stranded on the Calabrian coast and the particles stranded on the Sicilian coast was around 1.4, slightly underestimating the value obtained from the observations. This means that the model results are in good agreement with the empirical estimates.

## Discussion and conclusions

Considering that, during the early life stages, *P. noctiluca* spends most of the time on the surface layers without performing intense vertical migration^77^ and that during the maturity within the MS, the horizontal components of the transport are prevalent with respect to the vertical one, the approximation of a purely horizontal displacement of the particles was selected for all the numerical simulations. Specifically, in the MS the intense horizontal currents, up to 2 m s^−1^, are mainly barotropic and quite homogeneous along the vertical^52^. Therefore, the horizontal transport of the adult individuals, which perform intense nictemeral displacement, can be only partially affected by the vertical variability of the tidal flow, and the transport at the surface can be considered as an acceptable approximation for investigating the trapping processes within this area. Furthermore, the vertical components of the velocity fields computed by the hydrodynamic model were quite weak and around 1, 2 magnitude orders lower than the horizontal flow^52^, and, therefore, neglectable in the transport computation.

It should be noted that climatic factors are, however, expected to impact on the interannual fluctuations of *P. noctiluca* in this area. Indeed, the analysis of available long-term series of ocean dynamics and thermodynamics numerical model solutions^81,82^ and in situ and satellite observations^83^ have highlighted how interannual-to-decadal changes in the Mediterranean Sea and sub basins are a result of the decadal scale changes of the Northern Hemisphere atmospheric regimes, related to the variability of the North Atlantic Oscillation. Specifically, in the surroundings of the MS, the South-Eastern Tyrrhenian cyclonic Gyre^81^ and the Northern Sicily anticyclone^83^, at north, and the Atlantic Ionian Stream, at south, show noticeably amplitude variations, and a current reversal can take place in the Ionian Sea^81^.

The current knowledge of the interannual variability in the surroundings of the MS is hence mostly referred to a strengthening or weakening of the observed ocean circulation structures. Their effects on the highly variable occurrence of jellyfish blooms, over multiple years, should be carefully verified through ad hoc, high-resolution and long-period numerical simulations that should be supported by appropriate collections of blooms observations.

Although very different, the analyzed ecosystems of the Aeolian Islands and Messina Strait are complementary for the success of the *P. noctiluca* life cycle. Model results support the hypothesis of connectivity between these two ecosystems, especially in the first half of the year, suggesting that the coastal areas around the AIA serve as a potential source site for *Pelagia* in MS. Conversely, the MS is an area where immature specimens (20–30 mm) reach the average size of 40–50 mm in about three months. A growth rate of 0.28–0.30 mm d^−1^ and a rapid increase in biomass (0.73–1.13 g d^−1^) are greater than those recorded for other Mediterranean areas^21^. On the other hand, the MS does not seem to be optimal for *Pelagia'*s reproduction for two main reasons. The large short-term fluctuations in water temperatures (Fig. 5b), which are generally colder than the average of the Southern Tyrrhenian Sea, are mainly outside the optimal temperature range for *Pelagia* reproduction^79,84^. Moreover, the strong currents, far beyond the swimming capabilities of *Pelagia*^80^, do not favour large aggregations in which fertilization of broadcasted gametes is promoted. As a matter of fact, even if *P.noctiluca* specimens with mature gonads have been found, no planulae/ephyrae presence is reported by the authors who consider the MS a reproduction area^21,41^.

Massive blooms of *P. noctiluca* have been observed to occur at sites closest to the upper margins of marine canyons^9,38^, such as AIA basin^9^. The formation of these large aggregations facilitates the encounter of broadcasted gametes^85^, especially when thousands of specimens are in close contact. To enhance the success of sexual reproduction, the already observed frequent formation of individuals pairs during swarms^9^ was confirmed by us in two other configurations (Fig. S2).

Based on our observations and underwater videos showing new cohorts of planulae, ephyrae and juvenile medusae swarms in the Aeolian surface waters, we assume *Pelagia* reproduction in the AIA occurs from mid-winter to late spring (February-June). Reproduction may also occur in late autumn. Although *Pelagia* can reproduce throughout the year^21,40,86^, the main breeding seasons are spring and autumn at temperatures of 16–22 °C^79,84,87^. Water temperatures in mid-winter to late spring (17–22 °C) and autumn (18–26 °C) are characteristic of the AIA^88,89^ and are thus within the optimal range for *Pelagia* reproduction.

The AIA basin is an important breeding, spawning and nursery area for many commercially important pelagic and coastal fishes. It is a typical oligotrophic area^90,91^ with high species diversity^92–95^. The basin is outside the influence of upwelling, the thermocline is deeper, and the primary production and biomass of mesozooplankton are moderate^96–100^. These environmental and ecological characteristics, together with field observations, suggest that the archipelago may be a breeding site and an area for early larval development but the low productivity does not support the rapid growth of *P. noctiluca*.

The distribution and intensity of the *Pelagia* blooms depend largely on shelf topography, wind direction, and food concentration^101^. In the first half of the year, when northwesterly winds prevail, currents progress eastward from the AIA basin toward MS. As model simulations show, juvenile *Pelagia* specimens can take advantage of this direct connectivity link to reach MS from spawning grounds in just over 20–30 travel days on average. This is consistent with immature *Pelagia* specimens of about 20–40 mm bell diameter found in MS during the late winter-early spring months^21^.

Rosa et al.^21^ observed that the abundance of juvenile jellyfishes (20–30 mm) recorded from January to April at sampling sites in MS was only 4% of the total yearly *Pelagia* counts, indicating high larval mortality in winter. In addition, only jellyfishes greater than 20 mm were counted, as the smaller individuals were transparent and barely visible. Based on our data, we believe that the number of juvenile jellyfishes (up to 20–30 mm) entering MS in winter-spring is likely much higher than that already reported^21^. As shown by the winter-spring model simulations, particles are efficiently trapped during this period thanks to tidal back and forth movements^102^ and the presence of the residual recirculation cell near Cape Peloro.

From January to June, the number of particles entering the MS is higher than the number of particles moving from the MS to the STS (see “Living in the Strait (EXP2)” section Experiment 2). In contrast, the quantities are similar from June onwards, indicating a significant flux of particles from MS into the STS. Considering that the flow of seeded particles was homogeneous throughout the year (see “The numerical model” section Experiment 2) and that spawning occurs mainly in the first half of the year, the hypothesis that the northward flow drives juvenile *Pelagia* out of the MS from June to late summer is strengthened.

*P. noctiluca* was frequently observed at the entrance of the MS, in an area just north of Cape Peloro, which was probably related to the accumulation effect of the residual advective cyclonic eddy. Moreover, the large number of *P. noctiluca* along the Sicilian coast of the MS is closely associated with the effect of the residual circulation cells: once the *Pelagia* swarms enter the MS during the ebbing phase, they are driven northward by the main anticyclonic circulation or, alternatively, they can be trapped by the outflowing current along the Sicilian coast and move southward to accumulate within the Messina Harbour. Our results suggest that large numbers of *Pelagia* enter the MS from the STS when the winds are blowing from the NW and with ebbing currents. Their presence and abundance in the surface waters of the MS can change during the course of a day, from large densities at low tide to few specimens at high tide.

At the end of the spring, when the residual current is still low, and the number of particles in the MS reaches its maximum, the hydrodynamic regime in the Strait is dominated by tides^52^. The tidal dynamics in the MS, even if characterized by high instantaneous currents of up to 2.5 m s^−1^, has a relatively low residual circulation pattern, resulting in higher water residence times than in other seasons^52^. Comparing the obtained results with the numerical analysis, a coherence was observed between the periods with the highest particle arrival rate between the 150th and 175th day and the period with the highest particle residence times in MS, between the 130th and 180th day. These results further support the hypothesis that "*Pelagia* feed and grow in early summer in MS". In autumn and winter, surface circulation in the MS carries water masses into the Ionian and Tyrrhenian Seas, shortening the residence time of particles in MS^52^.

This study highlights the limitations of *Pelagia* (and other gelatinous zooplankton) distribution observations derived from fixed coastal sites. Physical factors such as winds influence the formation of aggregations and the occurrence of strandings and currents move the swarms along^103^. Thus, it happens that *P. noctiluca* swarms crossing the waters of MS are not recorded in shore-based observations, such as the bloom (about 600 ind. m^−3^) reported by fishermen on 28 April 2019, which was carried southward by the strong ebb current in the central part of MS. This strengthens the concept of the pelagic habitat of *P. noctiluca* and, consequently, the close correlation of this species with the circulation of water masses, upwelling systems and frontal zones^9,21,29^. *Pelagia* may indeed be passively transported by horizontal currents, so their temporal and spatial distribution results from the interplay of population dynamics and dispersal by currents^31^. In addition, some biological traits, such as vertical migration^104^ could improve the prediction of *Pelagia noctiluca* dispersal. It is difficult to assess these processes from observations alone, as neither in situ nor remote observations alone provide a continuous picture of circulation^31^. Therefore, we agree that modelling studies linking physical transport and monitoring of jellyfish outbreaks in coastal waters should be conducted^28^. *Pelagia* survival is lower in shallow waters because physical stresses damage their gelatinous bodies (bottom contact, wave action), daily migration is significantly disrupted, and predation by fish plays an important role^105^.

Stranding events on the Calabrian and Sicilian coasts of the MS are not a constant phenomenon with precise seasonal cycles that repeat themselves over the years. Rather, they must be considered as the result of various causes (biological, hydrological, meteorological) that determine more or less favourable periods for their occurrence. From an ecological point of view, though representing a net demographic and biomass loss due to the habitat boundary conditions, the massive stranding events and overall rates of stranding are accounted for in the adaptation strategy of the species to the habitat and can be sustained by the population of *Pelagia*, that receives in turn from the MS a sheltered and nutrient-rich environment during the central stages of the life cycle. The areas where *Pelagia* washed up in large numbers on the coasts are popular tourist areas. Our study agrees with the observations that the element of predictability of jellyfish strandings can help beach management authorities to plan and manage beach use and swimming activity when swarms of jellyfish appear near the shore^106^.

Finally, to close the biological cycle, we propose two different mechanisms of transporting the adult *P. noctiluca* individuals to the nursery in the Aeolian Islands. The hypothesis of Boero (in Sacchetti^37^), currently accepted also by many others^9,44,107^, seems plausible. Jellyfish are thought to migrate downward to reach intermediate water depths, possibly along canyon corridors. We believe that *P. noctiluca* individuals from MS are passively transported to deep Tyrrhenian waters through downwelling processes that occur primarily during well-stratified water conditions (summer and late summer). These processes are related to the interaction of internal solitary waves generated by tidal currents at the Ganzirri-P.ta Pezzo sill with seabed morphology (e.g. Gioia Tauro canyon and Calabrian slope), and propagating northwards in the Tyrrhenian Basin^108–110^. This phenomenon has been known for a long time, ever since Lohmann's^111^ valuable study of the distribution of appendicularians in the MS. Plankton species such as *Oikopleura longicauda* were used to observe and evaluate the deep downward currents. Later, this process was confirmed by the discovery of surface water indicator species, such as phytoplankton and chaetognaths, in the deep layers of the STS^57,112^. Marine canyons are known as "superhighways" due to the rapid circulation of water masses, sediments and organisms during active or passive movements from shallow to deeper waters and vice versa^9,36,113–115^. Canyons play an important role in structuring populations and life cycles of planktonic fauna^116^. On the northern margin of Sicily, a dense network of submarine canyons has been discovered in the depth range 80–2100 m, connecting the Sicilian and Tyrrhenian marine areas^117^. Intermediate waters may also provide an alternative, abundant crustacean resources for *P. noctiluca* (e.g., euphausiid crustaceans^114^). Thus, this jellyfish may spend the warmer months in deeper habitats along the continental slope with abundant food sources and invest more energy in future sexual reproduction through germ cell differentiation and gonad maturation^9^.

The other option of providing suitable transport of adult *P. noctiluca* individuals to the AIA would be by cyclonic currents in the south-eastern Tyrrhenian Sea. These could transport the individuals northward along the Italian coastline and then westward towards the AIA. The average summer geostrophic currents indicate possible routes of this type^49^. These currents are prone to instabilities and meanders and exhibit high interannual variability^49^. Our results can’t support or reject any of the two proposed mechanisms of transport towards the AIA. In fact both might be at work and due to considerable interannual variability of the Tyrrhenian circulation their contributions can be variable as well.

Our data support the hypothesis that there are two coupled (connected) systems, one of which favours *Pelagia*'s reproduction and serves as a nursery (in our case, the AIA), and the other favours its growth due to higher productivity (in our case, the MS). Therefore, we believe that the reproductive population of the AIA is not permanent but is renewed every year by individuals that have fed and grown rapidly in the MS and are transported by the ocean circulation. The most plausible is the transport by downwelling along the "corridors" of the canyons. This is precisely the phase that would complete the annual life cycle of *Pelagia*. Such a concept could have a broader impact if proven for other similar 'coupled' systems in the Mediterranean or elsewhere.

## Supplementary Information

Supplementary Video 1.

Supplementary Information 1.

Supplementary Information 2.

Supplementary Information 3.

Supplementary Information 4.
