# Supplementary material for: Observing and modeling long-term persistence of P. noctiluca in coupled complementary marine systems (Southern Tyrrhenian Sea and Messina Strait)
Source: Sci Rep. 2022 Sep 1;12:14905. doi: 10.1038/s41598-022-18832-2 (PMC9437060; doi:10.1038/s41598-022-18832-2)
Supplement: Supplementary file 2 — Supplementary Information 1. [file 41598_2022_18832_MOESM2_ESM.pdf]

Supplementary Material

Fig. S1: Stranding shares (%) per each coastal element along the Sicilian and Calabrian coasts of the Strait of Messina.

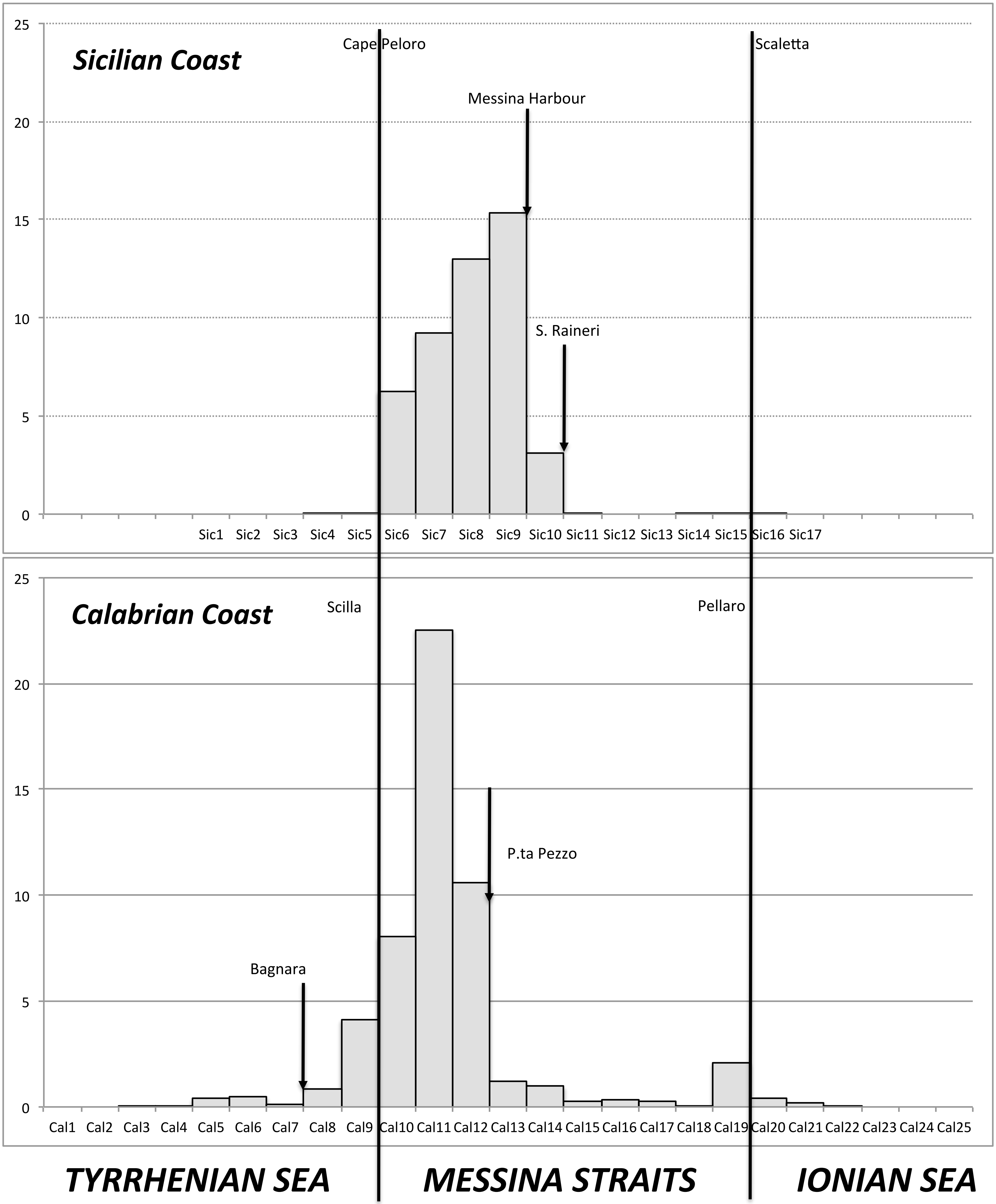

Sicilian Coast
Cape Peloro
Scaletta
Messina Harbour
S. Raineri
25
20
15
10
5
0
Sic1 Sic2 Sic3 Sic4 Sic5 Sic6 Sic7 Sic8 Sic9 Sic10 Sic11 Sic12 Sic13 Sic14 Sic15 Sic16 Sic17
Calabrian Coast
Scilla
Pellaro
P.ta Pezzo
Bagnara
Cal1 Cal2 Cal3 Cal4 Cal5 Cal6 Cal7 Cal8 Cal9 Cal10 Cal11 Cal12 Cal13 Cal14 Cal15 Cal16 Cal17 Cal18 Cal19 Cal20 Cal21 Cal22 Cal23 Cal24 Cal25
TYRRHENIAN SEA
MESSINA STRAITS
IONIAN SEA
