# Supplementary material for: Observing and modeling long-term persistence of P. noctiluca in coupled complementary marine systems (Southern Tyrrhenian Sea and Messina Strait)
Source: Sci Rep. 2022 Sep 1;12:14905. doi: 10.1038/s41598-022-18832-2 (PMC9437060; doi:10.1038/s41598-022-18832-2)
Supplement: Supplementary file 3 — Supplementary Information 2. [file 41598_2022_18832_MOESM3_ESM.pdf]

Supplementary material
Fig. S2

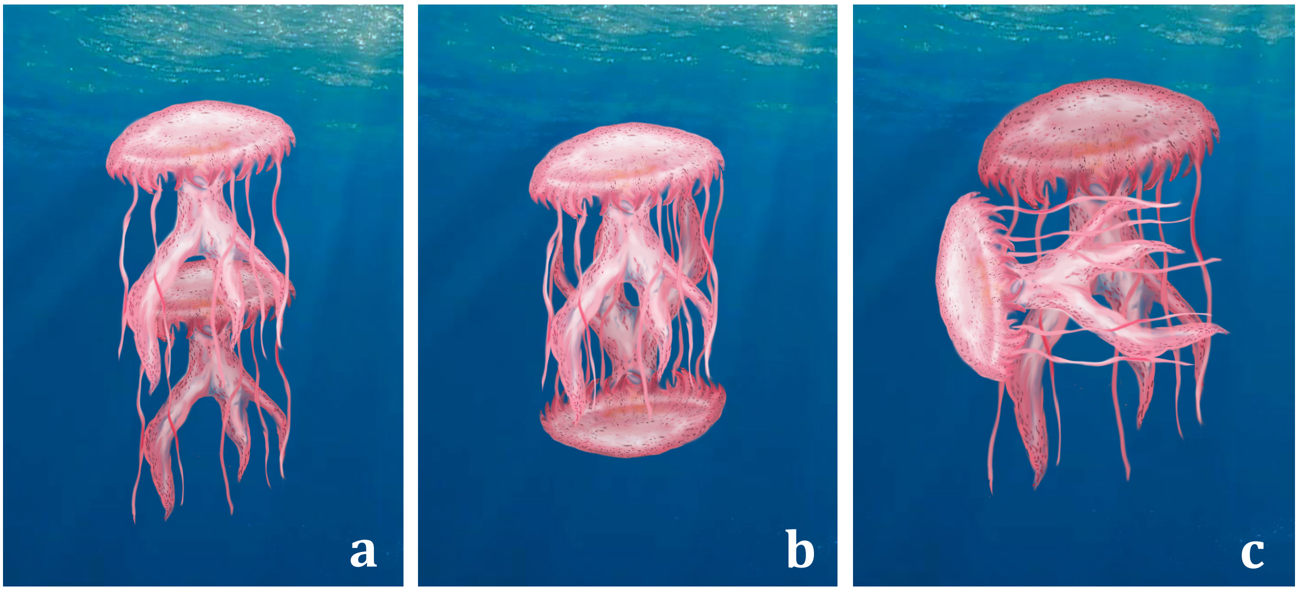

Fig. S2: Formation of individual pairs during *Pelagia noctiluca* swarms in the Aeolian Islands Archipelago: (a) the formation already reported[9] ; (b), (c) new formations.
