# Supplementary material for: Observing and modeling long-term persistence of P. noctiluca in coupled complementary marine systems (Southern Tyrrhenian Sea and Messina Strait)
Source: Sci Rep. 2022 Sep 1;12:14905. doi: 10.1038/s41598-022-18832-2 (PMC9437060; doi:10.1038/s41598-022-18832-2)
Supplement: Supplementary file 4 — Supplementary Information 3. [file 41598_2022_18832_MOESM4_ESM.docx]

**Supplementary Materials**

**V1: Videoclip Filicudi Island (Aeolian Archipelago), March 2009**

**P1** ÷**P6: Photos, March-June 2014**

Selected photos and videos reporting events of *Pelagia noctiluca* massive presence in the Aeolian Archipelago

Courtesy of Dario Lopes

Among the dozens of blooms observed in the last 15/18 years in the coastal waters of the Aeolian Islands, those certainly datable are here reported. They all refer to the period February / June.

* Filicudi, Apr 2008 <https://www.youtube.com/watch?v=iAUnbS9aRwc>

* **V1:** Filicudi, Mar 2009 Meduse Filicudi Marzo 2009.avi [57.41 MB]

**
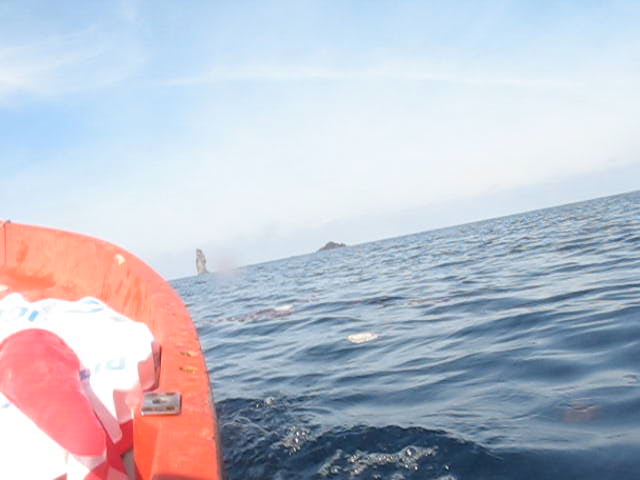
**

* Vulcano, Feb 2010 <https://www.youtube.com/watch?v=wXvKVGrBpuI>

* Filicudi, May 2010 <https://www.youtube.com/watch?v=nD4AHooBN6Q>

In the spring of 2014 the Aeolian Islands were affected by several very abundant blooms, documented with videos and photos both in Salina and Filicudi

* Filicudi Jun 2014 <https://www.youtube.com/watch?v=D9Htbe4LGnU>

* **P1:** Salina Marzo 2014.jpg [0.27 MB]

* **P2**: Filicudi Marzo 2014.jpg [0.15 MB]

* **P3:** Filicudi Maggio 2014.jpg [2.24 MB]

* **P4:** Filicudi Giugno 2014.png [0.38 MB]

* **P5:** Filicudi Giugno 2014 (2).png [0.25 MB]

* **P6:** Filicudi Giugno 2014 (3).png [0.24 MB]


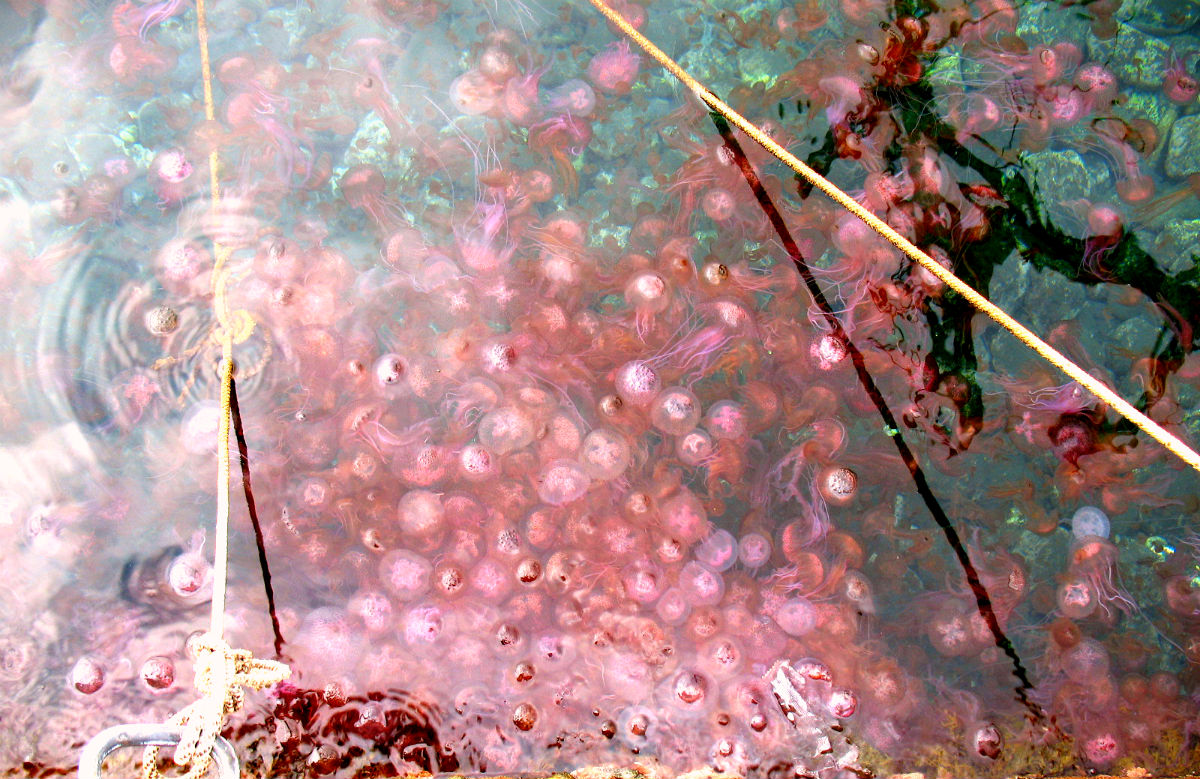


* P1: Salina Marzo 2014.jpg [0.27 MB]


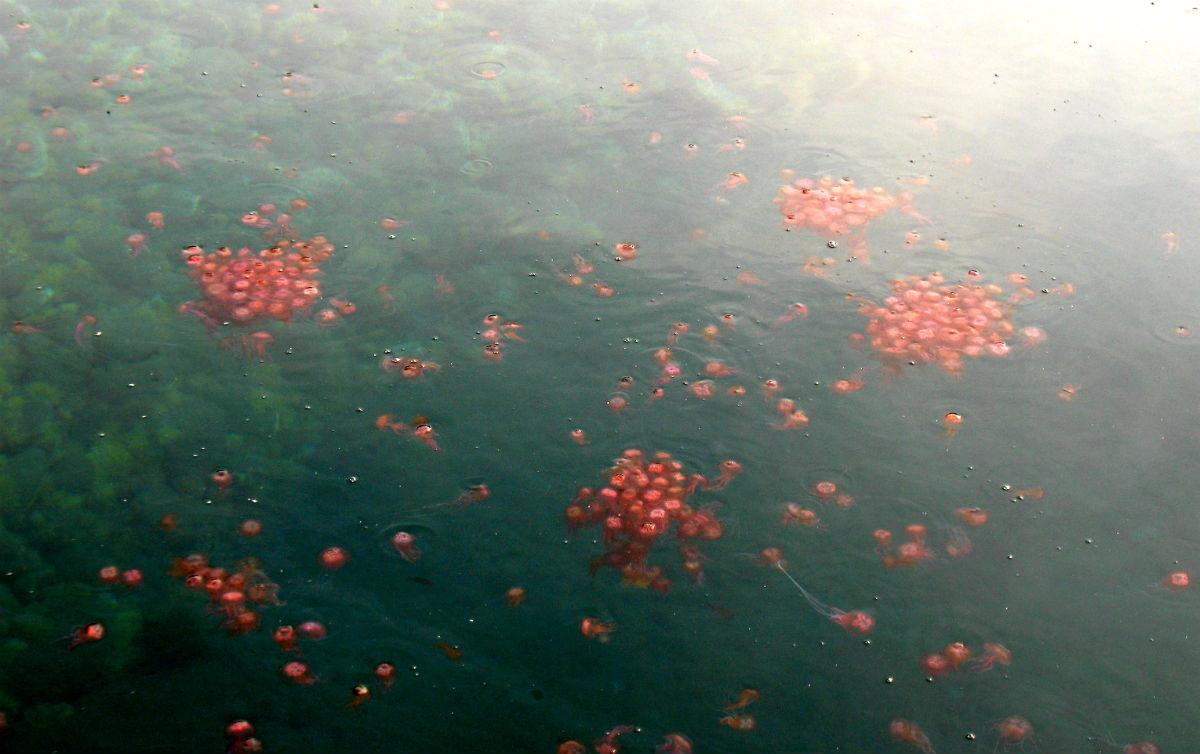


* P2: Filicudi Marzo 2014.jpg [0.15 MB]


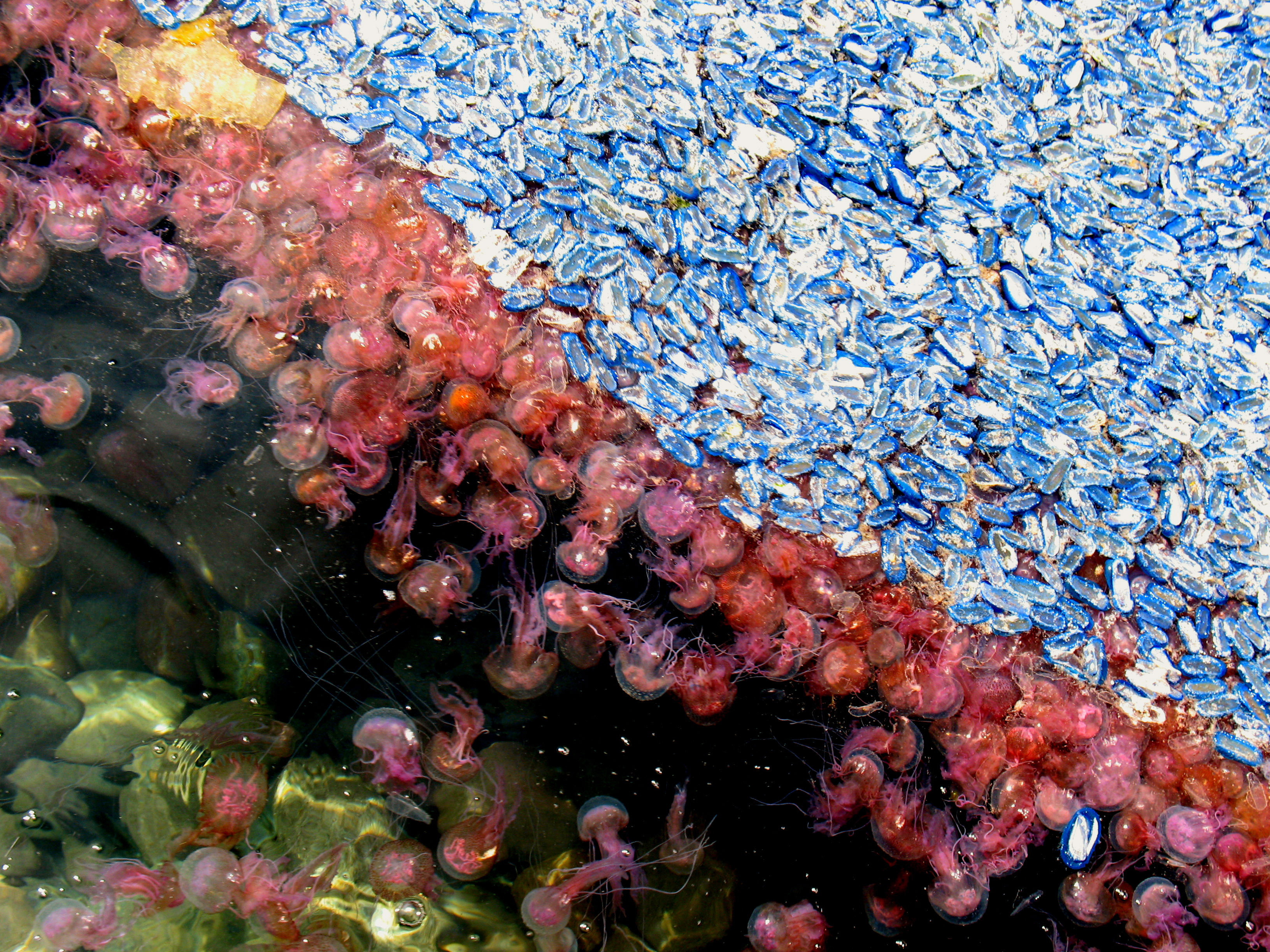


* P3: Filicudi Maggio 2014.jpg [2.24 MB]


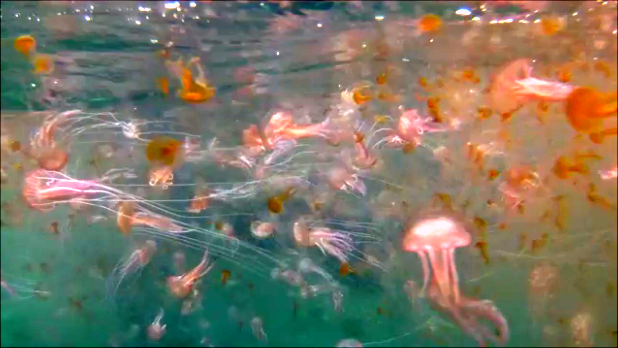


* P4: Filicudi Giugno 2014.png [0.38 MB]


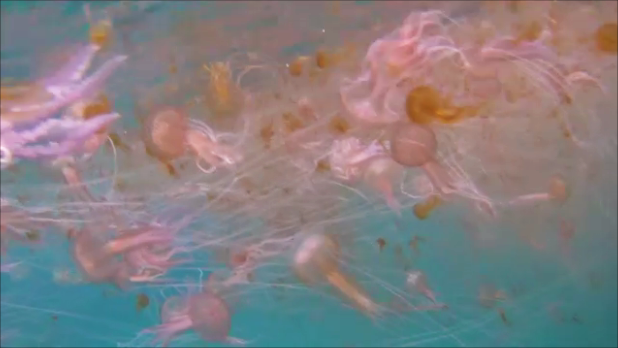


* P5: Filicudi Giugno 2014 (2).png [0.25 MB]


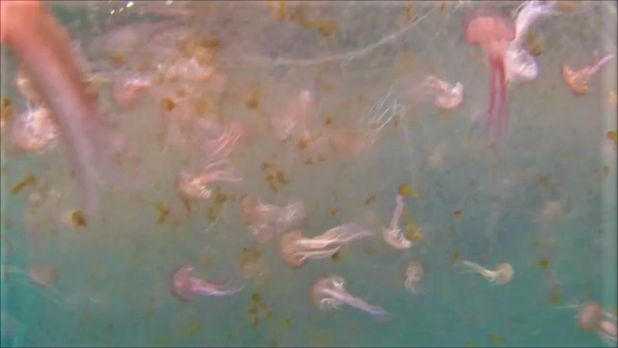


* P6: Filicudi Giugno 2014 (3).png [0.24 MB]
