# Supplementary material for: Observing and modeling long-term persistence of P. noctiluca in coupled complementary marine systems (Southern Tyrrhenian Sea and Messina Strait)
Source: Sci Rep. 2022 Sep 1;12:14905. doi: 10.1038/s41598-022-18832-2 (PMC9437060; doi:10.1038/s41598-022-18832-2)
Supplement: Supplementary file 5 — Supplementary Information 4. [file 41598_2022_18832_MOESM5_ESM.docx]

**Supplementary Note – Model implementation**

SHYFEM is a three-dimensional hydrodynamic model based on the finite element method [1] which resolves the shallow water equations integrated over each layer in their formulations with water levels and transports. The solved equation system for a single layer l reads as:

$$\frac{\partial U_{l}}{\partial t}+u_{l}\frac{\partial U_{l}}{\partial t}+v_{l}\frac{\partial U_{l}}{\partial t}+w_{l}\frac{\partial U_{l}}{\partial t}-{fV}_{l}={gh}_{l}\frac{\partial\zeta}{\partial x}-\frac{{gh}_{l}}{\rho_{0}}\frac{\partial}{\partial x}\int_{-H_{l}}^{\zeta} \rho^{'}dz+\frac{h_{l}}{\rho_{0}}\frac{{\partial p}_{a}}{\partial x}+\frac{1}{\rho_{0}}\left( \tau_{x}^{top\left( l \right)}-\tau_{x}^{bottom\left( l \right)} \right)+A_{H}\left( \frac{\partial^{2}U_{l}}{\partial x^{2}}+\frac{\partial^{2}U_{l}}{\partial y^{2}} \right)+\frac{\partial}{\partial z}\left( \frac{K_{l}}{h_{l}}\frac{\partial U_{l}}{\partial z} \right)+gh_{l}\frac{\partial\eta}{\partial x}-gh_{l}\beta\frac{\partial\zeta}{\partial x}$$

$\frac{\partial V_{l}}{\partial t}++u_{l}\frac{\partial V_{l}}{\partial t}+v_{l}\frac{\partial V_{l}}{\partial t}+w_{l}\frac{\partial U_{l}}{\partial t}-{fU}_{l}={gh}_{l}\frac{\partial\zeta}{\partial y}-\frac{{gh}_{l}}{\rho_{0}}\frac{\partial}{\partial y}\int_{-H_{l}}^{\zeta} \rho^{'}dz+\frac{h_{l}}{\rho_{0}}\frac{{\partial p}_{a}}{\partial y}+\frac{1}{\rho_{0}}\left( \tau_{y}^{top\left( l \right)}-\tau_{y}^{bottom\left( l \right)} \right)+A_{H}\left( \frac{\partial^{2}V_{l}}{\partial x^{2}}+\frac{\partial^{2}V_{l}}{\partial y^{2}} \right)+\frac{\partial}{\partial z}\left( \frac{K_{l}}{h_{l}}\frac{\partial V_{l}}{\partial z} \right)+gh_{l}\frac{\partial\eta}{\partial y}-gh_{l}\beta\frac{\partial\zeta}{\partial y}$

$\frac{\partial\zeta}{\partial t}+\sum_{l} \frac{\partial U_{l}}{\partial x}+\sum_{l} \frac{\partial U_{l}}{\partial x}=0$ (A1)

where $l$ indicates the vertical layer, ($U_{l},V_{l}$) the horizontal transport components in x and y directions for each layer, ${(u}_{l},v_{l}, w_{l})$the velocity components, $f$ the Coriolis parameter, $p_{a}$the atmospheric pressure,$g$ the gravitational constant, $\zeta$the sea level, $\rho_{0}$the standard water density, ${\rho=\rho^{'}+\rho}_{0}$the water density, $\tau$the internal stress term at the top and bottom of each layer, $h_{l}$the layer thickness, $H_{l}$ the depth of the bottom of the layer $l$, $A_{H}$the horizontal eddy viscosity estimated following the Smagorinsky parameterization [2].

The GOTM (General Ocean Turbulence Model) turbulence closure model described in [3] was used for the computation of the vertical viscosity $K_{l}$. Momentum exchanges across the model layers are accounted by computing both the advective contribution and the vertical constituents of the diffusive terms $\frac{\partial}{\partial z}\left( \frac{K_{l}}{h_{l}}\frac{\partial U_{l}}{\partial z} \right) and \frac{\partial}{\partial z}\left( \frac{K_{l}}{h_{l}}\frac{\partial V_{l}}{\partial z} \right)$.

Equilibrium tidal potential ($\eta$) and load tides are included as model forcing. The term $\eta$ is computed as the sum of the tidal potential of each tidal constituent multiplied by the frequency-dependent elasticity factor [4], whereas the factor $\beta$ accounts for the effects of load tides [5].

Wind and bottom friction terms, corresponding to the boundary conditions of the stress terms ($\tau_{x},\tau_{y}$), are defined as:

$\tau_{x}^{surface}=c_{D}\rho_{a}{wi}_{x}\sqrt{{wi}_{x}^{2}+{wi}_{y}^{2}}$

$\tau_{x}^{bottom}=c_{B}\rho_{0}u_{L}\sqrt{u_{L}^{2}+v_{L}^{2}}$

$\tau_{y}^{surface}=c_{D}\rho_{a}{wi}_{y}\sqrt{{wi}_{y}^{2}+{wi}_{y}^{2}}$

$\tau_{y}^{bottom}=c_{B}\rho_{0}v_{L}\sqrt{u_{L}^{2}+v_{L}^{2}}$ (A2)

with $c_{D}$ as the wind drag coefficient, $c_{B}$ the bottom friction coefficient, $\rho_{a}$ the air density (${wi}_{x}, {wi}_{y}$) the wind velocity components and ($u_{L},v_{L}$) the bottom velocity components.

The hydrodynamic model is coupled with a particles trajectories module (PTM) that solves the advection and diffusion equation in a Lagrangian framework of reference:

$\frac{\partial x}{\partial t}=u_{a}+u_{d}$

$\frac{\partial y}{\partial t}=v_{a}+v_{d}$ (A3)

where $u_{a}$, $v_{a}$ are the advective velocities components and $u_{d}$, $v_{d}$ are the diffusive velocities components in x and y directions, respectively. The $u_{d}$ and $v_{d}$ components are computed using a random walk technique on the basis of the [6] study, with turbulent diffusion coefficients obtained using the Smagorinsky parameterization [2].

The components $u_{a}$ and $v_{a}$are expressed as:

$u_{a}=u_{1}+\alpha_{w}w_{x}$ (A4)

$v_{a}=v_{1}+\alpha_{w}w_{x}$

where $u_{1}$, $v_{1}$are the horizontal velocities computed by the hydrodynamic model for the first vertical layer; $w_{x}$, $w_{x}$ are the wind speed components; $\alpha_{w}$ is the wind transport factor set to 0.004 in this application. The PTM equation system is solved on the same numerical mesh used by the hydrodynamic model. Details of the numerical treatment are reported in [7].

SHYFEM has been applied to the whole Messina Strait and part of the Tyrrhenian and Ionian Sea to reproduce the surface water circulation and the related transport during the biennial 2014 and 2015. The numerical computation is carried out on the same finite element mesh adopted in [8] constituted by around 23000 nodes and 45000 triangular elements with a spatial resolution varying between 50 m for the inner part of the Strait and up to 3 km for the far field (Fig. 4 of the main text). Two open boundaries were selected in correspondence of the southern and northern borders of the mesh located approximately in the Northern Ionian and in the Southern Tyrrhenian Sea, respectively. A Dirichlet open boundary condition was adopted, with water levels imposed and current velocities freely computed by the model along the open boundaries. At the closed boundaries a full slip condition was imposed with the normal velocity set to zero and the tangential velocity computed by the model.

The vertical direction was discretized by 30 uneven z-levels with layer thicknesses varying from 5 m, for the uppermost layer, to 800 m for the ocean seafloor and following an ad hoc step distribution with by maxima layers density in the first 100 meters of water depth.

Forcing and boundary conditions necessary to reproduce the water circulation and the induced transport in the study area were constituted by oceanographic and atmospheric data obtained from different sources.

Specifically, the atmospheric data necessary to compute the heat fluxes and momentum transfer were provided by the meteorological prediction system of the European Centre for Medium-Range Weather Forecasts (ECMWF, https://www.ecmwf.int). These data include the air temperature, precipitation, evaporation rate, humidity, atmospheric pressure and wind components with a temporal and spatial resolution of 6 hours and approximately 12 km at these latitudes.

Ocean data were provided by the Tyrrhenian Sicily Channel sub-Regional Model (TSCRM) an operational forecasting system based on a full three-dimensional implementation of the Princeton Ocean Model [9] applied to the whole Tyrrhenian Sea and Sicily Channel and already used and tested in previous applications with SHYFEM [8, 10, 11]

. The obtained dataset constituted by 6 hourly fields of water temperature, salinity and water levels was used both as open boundary conditions and for nudging procedure.

Tidal forcing was obtained from the tidal model TPXO7.2 and consisted in hourly time series of tidal elevation extracted along the open boundaries of the model mesh. In particular, the tidal elevations data were reconstructed using OTIS (OSU Tidal Inversion Software), a tidal data inversion package [12, 13] taking into account eight primaries (M2, S2, N2, K2, K1, O1, P1, Q1), two long period (Mf, Mm) and 3 non-linear (M4, MS4, MN4) harmonic constituents. The water levels boundary conditions were reconstructed for each node of the mesh open boundaries by summing the astronomical tides to the water elevation data provided by the TSCRM.

Ocean, tidal and atmospheric data for the years 2014 and 2015 were used as forcing for a 2-year long simulation run using the same model parametrization and setup adopted in [8]. The hourly fields of computed surface current velocities obtained from the hydrodynamic simulation were used as inputs data for the PTM to investigate both the connectivity pattern between the jellyfish spawning regions located in the Tyrrhenian Sea and the Messina Strait and the transport dynamics inside the Strait itself.

REFERENCES

1. Umgiesser, G., Canu, D.M., Cucco, A. & Solidoro, C. A finite element model for the Venice Lagoon. Development, set up, calibration and validation. *J. Mar. Sys.* **51**, 123-145 (2004).
2. Smagorinsky, J. Some historical remarks on the use of non-linear viscosities, In: Large Eddy Simulation of Complex Engineering and Geophysical Flows, edited by B. Galperin and S. A. Orszag, pp. 3–36, Cambridge Univ. Press, Cambridge, U. K. (1993)
3. Burchard, H.; Petersen, O. Models of turbulence in the marine environment—A comparative study of two-equation turbulence models. *J. Mar. Syst.* **21**, 29–53 (1999).
4. Kantha, L.H. and Clayson, C.A., Numerical models of oceans and oceanic processes. *Int. Geophys.*, **66**, (2000).
5. Kantha, L.H., Barotropic tides in the global oceans from a nonlinear tidal model assimilating altimetric tides 1. Model description and results. *J. Geophys. Res.* **100** (C12), 283–309 (1995).
6. Fischer, H. B., List, J. E., Koh, C. R., Imberger, J., & Brooks, N. H. Mixing in inland and coastal waters. Academic press, (1979)
7. Cucco, A., Quattrocchi, G., Satta, A., Antognarelli, F., de Biasio, F., Cadau, E., Umgiesser, G., & Zecchetto, S. Predictability of wind-induced sea surface transport in coastal areas. *J. Geophys. Res.*: Oceans, **121**(8), 5847–5871. (2016). <https://doi.org/10.1002/2016JC011643>
8. Cucco, A, Quattrocchi, G, Olita, A, Fazioli, L, Ribotti, A, Sinerchia, M, Tedesco, C, Sorgente, R. Hydrodynamic modelling of coastal seas: the role of tidal dynamics in the Messina Strait, Western Mediterranean Sea. *Nat. Hazards Earth Syst. Sci.* **16**, 1553-1569 (2016).
9. Mellor, G. An equation of state for numerical models of oceans and estuaries. *J. Atmos. Ocean. Technol.* **8**, 609–611, (1991).
10. Cucco, A., Sinerchia, M., Ribotti, A., Olita, A., Fazioli, L., Perilli, A., Sorgente, R. A high-resolution real-time forecasting system for predicting the fate of oil spills in the Strait of Bonifacio (western Mediterranean Sea). *Mar. Pollut. Bull.* **64**(6), 1186–1200, (2012).
11. Ribotti, A., F Antognarelli, A Cucco, M Falcieri, L Fazioli, C Ferrarin, A Olita, …& Sorgente R. (2019). An Operational Marine Oil Spill Forecasting Tool for the Management of Emergencies in the Italian Seas. *J. Mar. Sci. Eng.*, 7(1), 1; https://doi.org/10.3390/jmse7010001
12. Egbert, G. D., Bennett, A. F. and Foreman, M. G. G. TOPEX/POSEIDON tides estimated using a global inverse model, *J. Geophys. Res.*, 99 (C12), 24821– 24852, (1994) doi:10.1029/94JC01894.
13. Egbert, G.D. and Erofeeva, S.Y. Efficient Inverse Modeling of Barotropic Ocean Tides. *J. Atmos. Oceanic Technol.*, **19**, 183–204, (2002) https://doi.org/10.1175/1520-0426(2002)019<0183:EIMOBO>2.0.CO;2
